# Integrative Multiomics Mendelian Randomization Prioritizes Endoplasmic Reticulum Stress‐Related Genes Associated With Gastric Cancer Risk

**DOI:** 10.1155/humu/3845963

**Published:** 2026-10-27

**Authors:** Dong Yang, Yuanlin Liu, Xiangyu Meng, Chao Wang, Zhiyong Yu, Tao Zhang

**Affiliations:** ^1^ Department of Gastrosurgery Liaoning Cancer Hospital and Institute Shenyang China lnszl.com; ^2^ Department of General Surgery Yingkou Central Hospital Yingkou China

**Keywords:** colocalization, endoplasmic reticulum stress, gastric cancer, Mendelian randomization analysis, multiomics, single-cell RNA sequencing

## Abstract

**Background:**

Emerging research indicates connections between endoplasmic reticulum (ER) stress and gastric cancer (GC), but related genetic factors remain unclear. This study is aimed at prioritizing ER stress‐related genes associated with GC risk using multiomics data.

**Methods:**

We integrated GC GWAS data from FinnGen (discovery) and United Kingdom Biobank (replication) cohorts with QTL datasets, including mQTLs, eQTLs, and pQTLs. Summary‐data‐based Mendelian randomization (SMR) and colocalization analyses were performed to prioritize candidate genes, followed by characterization using transcriptomic profiling (GSE118916), methylation‐expression correlation analysis (TCGA‐STAD), and single‐cell RNA‐seq analysis (GSE270680).

**Results:**

SMR analyses identified 83 mQTL, 10 eQTL, and 2 pQTL signals associated with GC risk. Among these, colocalization evidence was observed for 47 mQTL, 5 eQTL, and 2 pQTL signals. Transcriptomic analysis showed that *PCSK6*, *YOD1*, and *RNF5* exhibited expression changes directionally consistent with their SMR associations with GC risk, whereas *CTNNBIP1* showed an inconsistent expression pattern. *PCSK6* was supported by mQTL, eQTL, and mQTL‐eQTL evidence but lacked colocalization. *YOD1*, identified through pQTL‐SMR, showed strong colocalization evidence (PPH4 = 0.95). *RNF5* was highlighted via eQTL‐SMR but lacked colocalization support. *CTNNBIP1* was supported by mQTL, eQTL, and mQTL‐eQTL evidence and colocalization, with a significant methylation‐expression correlation observed in TCGA‐STAD. Single‐cell RNA‐seq revealed that *PCSK6* was predominantly expressed in fibroblasts, *YOD1* in epithelial cells and mast cells, and *RNF5* broadly across fibroblasts, epithelial cells, and selected immune cells.

**Conclusions:**

This multiomics study prioritized *PCSK6*, *YOD1*, and *RNF5* as ER stress‐related candidate genes showing genetic associations with GC risk and directionally consistent expression patterns, with single‐cell analysis indicating distinct cell‐type‐specific expression patterns. These findings offer useful context for future functional and mechanistic investigations of the prioritized candidate genes.

## 1. Introduction

Gastric cancer (GC) is one of the most prevalent malignancies worldwide and remains the fourth leading cause of cancer‐related mortality [1]. Previous global estimates reported approximately 990,000 individuals were diagnosed with GC and nearly 738,000 deaths were attributed to this disease annually [2]. Several factors have been reported to influence GC susceptibility, including genetic predisposition, dietary habits, alcohol intake, tobacco use, as well as infections caused by *Helicobacter pylori* and the Epstein–Barr virus [1]. Despite advances in diagnostic techniques and treatment options, such as surgery, chemotherapy, and targeted therapies, the overall prognosis of GC remains poor, particularly among patients with advanced‐stage disease, for whom the 5‐year survival rate remains below 30% [3]. Therefore, a deeper understanding of the genetic and molecular factors associated with GC development is crucial for identifying potential biomarkers for early diagnosis and prioritizing candidate molecules for future investigation.

Accumulating evidence indicates that genetic alterations and dysregulated molecular pathways play important roles in GC biology. Among these mechanisms, endoplasmic reticulum (ER) stress and the unfolded protein response (UPR) have been implicated in several malignant phenotypes of GC, including enhanced cell proliferation, angiogenesis, metastatic spread, tumor microenvironment (TME) modulation, and treatment resistance [4]. Several ER‐associated molecular chaperones have also been implicated in GC pathogenesis. GRP78, which is highly expressed in gastric tumors, significantly promotes GC cell proliferation, antiapoptosis, invasion, and treatment resistance [5]. Similarly, the ER chaperone GRP94 has been associated with GC progression, growth, invasion, and metastatic potential [6]. Additionally, reduced SERCA3 expression in GC has been inversely correlated with tumor invasiveness, distant metastases, and advanced tumor‐node‐metastasis (TNM) stage [7]. However, many existing studies of ER stress in GC have relied on observational designs or small‐scale transcriptomic profiling, which may be affected by reverse causality, confounding factors, and limited reproducibility. Furthermore, findings derived from single‐omics analyses may provide only a partial view of the molecular features associated with ER stress in GC and often lack support across multiple molecular layers. Consequently, the genetic factors and molecular networks specifically linking ER stress to GC risk have not been systematically characterized, highlighting the necessity for integrated multiomics approaches to prioritize ER stress‐related candidate genes and provide complementary molecular evidence.

Summary‐data‐based Mendelian randomization (SMR) provides a genetic framework for integrating publicly available genome‐wide association study (GWAS) summary statistics with quantitative trait loci (QTL) data for DNA methylation, gene expression, and protein abundance to prioritize genes whose genetically regulated molecular features may be associated with disease phenotypes [8]. Recently, the integration of SMR, the Heterogeneity in Dependent Instruments (HEIDI) test, and Bayesian colocalization analysis has been increasingly used in multiomics MR studies [9, 10]. This combined approach helps assess whether QTL and GWAS signals are likely to share the same genetic variant and helps distinguish shared genetic signals from associations attributable to linkage disequilibrium (LD) [8]. In cancer research, including GC, the integration of SMR, HEIDI testing, and colocalization has provided a useful strategy for prioritizing genetically supported candidate genes and molecular traits across different cancer types and omics layers [11–13]. Recent cancer studies integrating bulk transcriptomic datasets with single‐cell transcriptomic analyses have further highlighted the value of multilayer evidence integration for characterizing tumor heterogeneity and prioritizing candidates [14]. Applying this framework to ER stress may therefore facilitate the systematic prioritization of genetically supported ER stress‐related genes associated with GC.

In this study, the combined use of SMR, HEIDI, and colocalization analysis provided a framework for prioritizing ER stress‐related genes whose genetically regulated molecular traits were associated with GC risk, while distinguishing shared genetic signals from associations attributable to LD. By integrating SMR analysis, Gene Expression Omnibus (GEO) transcriptomic profiling, The Cancer Genome Atlas (TCGA) methylation‐expression correlation analysis, and single‐cell transcriptomic data, this study systematically evaluated ER stress‐related candidate genes across multiple dimensions, including genetic associations, methylation‐expression correlations, transcriptional alterations, and cell‐type‐specific expression patterns. Our findings provide supporting evidence for the prioritization of candidate genes for subsequent functional and mechanistic investigations.

## 2. Materials and Methods

### 2.1. Study Design

In our study, we identified instrumental variables associated with ER stress‐related molecular traits across three omics layers: DNA methylation, gene expression, and protein abundance. To explore their potential genetic associations with GC risk, we conducted SMR analyses at each molecular layer. The primary dataset for discovery was derived from the FinnGen R10 cohort, and for the replication dataset, we utilized GC GWAS data from the United Kingdom Biobank cohorts. To strengthen the interpretation of SMR findings, HEIDI testing was used to assess heterogeneity potentially attributable to LD, whereas colocalization analysis was performed to evaluate whether the QTL and GC GWAS signals were likely to share the same genetic variant. By integrating multiomics SMR analysis with HEIDI and colocalization assessment, we prioritized ER stress‐related candidate genes with genetic evidence of association with GC risk and subsequently performed further characterization using GEO transcriptomic expression profiles, TCGA methylation‐expression correlation analysis, and single‐cell transcriptomic data. Figure 1 presents an overview of the study framework and the main analytical procedures. The study was reported in accordance with the STROBE‐MR reporting guidelines [15]. All summary statistics used in the SMR analysis were obtained from previously published studies or publicly available resources with appropriate ethical approval.

**Figure 1:**
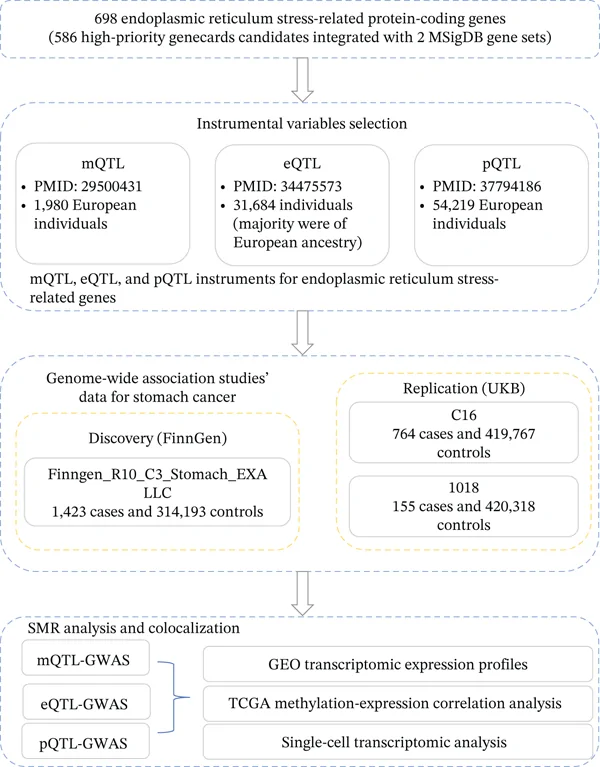
Flowchart of the analyses performed. The analytical workflow began with the identification of endoplasmic reticulum (ER) stress‐related genes. A summary‐data‐based Mendelian randomization (SMR) approach was then employed by integrating gastric cancer (GC) genome‐wide association study (GWAS) data from the FinnGen discovery cohort with quantitative trait loci (QTL) data for DNA methylation (mQTLs), gene expression (eQTLs), and protein abundance (pQTLs). Significant associations were further evaluated in independent GC GWAS datasets from the UK Biobank replication cohorts. Candidate signals were assessed using SMR‐multi, the HEIDI test, and colocalization analysis to prioritize genes with more consistent genetic evidence and reduce the likelihood of linkage disequilibrium‐driven associations. Prioritized genes were further characterized using external transcriptomic analysis in GSE118916, methylation‐expression correlation analysis in TCGA‐STAD, and single‐cell RNA‐seq analysis in GSE270680.

### 2.2. Data Sources

We obtained the GWAS summary statistics for GC from the FinnGen R10 cohort (https://storage.googleapis.com/finngen-public-data-r10/summary_stats/finngen_R10_C3_STOMACH_EXALLC.gz), including 1423 cases and 314,193 controls. GC cases in the FinnGen cohort were defined using registry‐based diagnostic codes, including ICD‐10 code C16 and the corresponding ICD‐8/9 codes, together with ICD‐O‐3 topography code C16 from the Finnish Cancer Registry. For replication, we used two independently defined GC phenotypes from the United Kingdom Biobank. The first was an ICD‐10‐based phenotype for malignant neoplasm of the stomach (C16), comprising 764 cases and 419,767 controls. The second was based on the United Kingdom Biobank self‐reported cancer code 1018 (stomach cancer), comprising 155 cases and 420,318 controls. Notably, the case numbers in the United Kingdom Biobank cohorts were smaller than in the FinnGen discovery cohort, which may limit the statistical power for replication. Detailed information on all GWAS datasets used in this study is summarized in Table S1.

Using the keyword “endoplasmic reticulum stress”, we retrieved 2764 genes from the GeneCards database (https://www.genecards.org). After restricting the results to protein‐coding genes and ranking them by relevance score, we retained the top 25% of protein‐coding genes, corresponding to 586 candidate genes with a relevance score threshold of 2.104952. To further expand the gene set, we incorporated two ER stress‐related gene sets from the Molecular Signatures Database (MSigDB), namely GOBP_RESPONSE_TO_ENDOPLASMIC_RETICULUM_STRESS and GOBP_REGULATION_OF_RESPONSE_TO_ENDOPLASMIC_RETICULUM_STRESS. The union of these sources yielded a final set of 698 ER stress‐related genes for subsequent SMR analysis. The original GeneCards‐derived genes, the high‐priority GeneCards candidates, and the final integrated ER stress‐related gene set are provided in Table S2.

We acquired blood eQTL data from the eQTLGen consortium, encompassing gene expression profiles for 31,684 individuals [16]. The mQTL data were obtained through a meta‐analysis of two European cohorts: the Brisbane Systems Genetics Study, which involved 614 participants, and the Lothian Birth Cohorts, which included 1366 participants [17]. Additionally, pQTL data were retrieved from a study by Sun BB et al., which comprised 54,219 participants from the United Kingdom Biobank [18]. Detailed information on all QTL datasets used in this study is summarized in Table S1.

### 2.3. Summary‐Data‐Based MR Analysis

SMR was performed to evaluate the potential genetically supported associations between genetically regulated molecular traits of ER stress‐related genes, including DNA methylation, gene expression, and protein abundance, and GC risk. Within the SMR framework, the three core assumptions of MR were carefully considered. First, for the relevance assumption, genome‐wide significant *cis*‐QTLs located within ± 1 Mb of the target gene were selected as instrumental variables. This stringent selection strategy helped ensure strong associations between the selected *cis*‐QTLs and corresponding molecular traits, thereby reducing the likelihood of weak‐instrument bias. Second, for the independence assumption, only *cis*‐acting variants near the target gene were used. Compared with *trans*‐acting variants, *cis*‐QTLs are more directly involved in local molecular regulation and are less likely to be influenced by systemic environmental or behavioral confounders. Third, for the exclusion restriction assumption, SMR does not rely on the conventional stringent LD clumping strategy commonly used in multi‐instrument MR. Instead, it leverages the local LD structure within the *cis*‐region and evaluates potential LD‐driven associations using the HEIDI test and, where applicable, the SMR‐multi extension.

This approach enhanced statistical power when using top *cis*‐QTLs compared with the conventional MR analysis [19]. The selection of top *cis*‐QTLs was based on a genomic window spanning ± 1000 kb around the gene of interest, coupled with a stringent significance threshold of *p* < 5.0 × 10^−8^ [20, 21]. In the standard single‐SNP SMR analysis, the top genome‐wide significant *cis*‐QTL within ± 1 Mb of the target gene was selected as the primary instrumental variable to prioritize locally acting molecular QTLs. SNPs with allele frequency differences greater than 0.2 across the LD reference panel, QTL datasets, and outcome GWAS datasets were excluded to avoid potential bias from population structure differences.

Besides exploring the potential genetically supported associations between molecular QTLs (mQTLs, eQTLs, and pQTLs) and GC risk, we further explored cross‐omics genetic associations, treating mQTLs as exposures and eQTLs as outcomes, as well as eQTLs as exposures and pQTLs as outcomes.

Building upon the standard SMR framework, we further utilized a multi‐SNP‐based SMR approach (‐‐smr‐multi), which incorporates all SNPs within a specific QTL probe window (default ± 500 kb) that satisfy two criteria: *p* < 5.0 × 10^−8^ and LD *r* ^2^ < 0.9 with the top associated SNP [22]. The SMR‐multi method integrates multiple incompletely linked QTL signals within the *cis*‐region to improve statistical power and enhance inference on genetically regulated molecular traits. Compared with traditional single‐SNP SMR methods, SMR‐multi allows the utilization of multiple SNP signals within the *cis*‐region, while accounting for correlations among these signals. Associations supported by both single‐SNP and multi‐SNP SMR analyses were prioritized.

Subsequently, the HEIDI test was applied to assess heterogeneity potentially attributable to LD and to help distinguish shared genetic signals from associations driven by distinct linked variants. Findings meeting the criteria (*P* − *S* *M* *R* < 0.05, *P* − *S* *M* *R* − *m* *u* *l* *t* *i* < 0.05, and *P* − *H* *E* *I* *D* *I* > 0.05) were subsequently subjected to colocalization analysis and a composite assessment involving eQTLs, mQTLs, and pQTLs.

### 2.4. Colocalization Analysis

Colocalization analysis was used to identify whether ER stress‐related *cis*‐QTLs (including eQTLs, mQTLs, and pQTLs) and GC GWAS signals were likely to share a common causal variant. In the colocalization analysis, five distinct posterior probabilities were reported, corresponding to five mutually exclusive hypotheses: (1) neither trait in the region is genetically associated with the SNP (H0); (2) only the first trait is genetically associated with the SNP (H1); (3) only the second trait is genetically associated with the SNP (H2); (4) both traits are associated with the SNP, but through distinct causal variants (H3); and (5) both traits are associated with the SNP and share a common causal variant (H4). Colocalization analysis was conducted on SNPs located within ± 1000 kb of the top *cis*‐QTL [23, 24].

Although a PPH4 (posterior probability of H4) ≥0.8 is generally regarded as strong Bayesian evidence supporting colocalization, Breen et al. observed that many loci with PPH4 ≥ 0.5 also appear qualitatively consistent with colocalization phenomena [25, 26]. Given the exploratory nature of the present study, we therefore used PPH4 > 0.5 as the posterior probability threshold to increase the sensitivity for detecting potential colocalization signals, while recognizing that this threshold is more permissive than the conventional PPH4 > 0.8 criterion. Accordingly, evidence from colocalization analysis was classified into two confidence levels: loci with PPH4 > 0.8 were considered to provide strong colocalization evidence, whereas loci with 0.5 < PPH4 ≤ 0.8 were considered to provide suggestive colocalization evidence warranting further investigation rather than definitive evidence of colocalization.

All statistical analyses were performed using R (Version 4.3.0). The R packages “ggplot2” and “ggrepel” were used to create Manhattan plots, and “forestplot” was utilized to generate forest plots. Visualization code for SMR locus plots and effect plots (SMRLocusPlot and SMREffectPlot) was adapted from the original SMR implementation described by Zhu et al. [19].

### 2.5. Functional Enrichment Analysis

To evaluate whether the gene filtering strategy retained the core biological features of ER stress, functional enrichment analysis was performed separately for the original gene set (2764 genes) and the final 698‐gene set used for SMR analysis, using the R package “clusterProfiler”. Gene Ontology (GO) enrichment was performed for the biological process, molecular function, and cellular component categories, and Kyoto Encyclopedia of Genes and Genomes (KEGG) pathway enrichment was performed with disease‐related pathways excluded. Enrichment *p* values were adjusted using the Benjamini–Hochberg method, and terms with adjusted *p* < 0.05 were considered significantly enriched.

### 2.6. External Assessment in Gastric Tissue Transcriptome Dataset

To examine whether the expression patterns of SMR‐prioritized candidate genes were directionally consistent with their genetically predicted associations with GC risk, gene expression profiles were obtained from the GEO database (https://www.ncbi.nlm.nih.gov/geo/). The GSE118916 dataset, generated on the GPL15207 platform, includes 15 gastric tumor samples and 15 normal stomach tissue samples. Raw microarray data were processed by mapping probes to gene symbols, removing probes without corresponding gene annotations, and averaging expression values when multiple probes mapped to the same gene.

Differential expression analysis between GC and normal tissues was performed using the R package “limma”, and genes with *p* < 0.05 were considered differentially expressed. For visualization of candidate gene expression, boxplots were generated using the R package “ggplot2”, and the corresponding *p* values displayed in the figures were calculated using the Wilcoxon rank‐sum test.

### 2.7. Correlation Analysis of DNA Methylation and Gene Expression in TCGA‐STAD

To further evaluate the association between specific CpG site methylation and target gene expression, we obtained paired bulk transcriptomic and DNA methylation data from the stomach adenocarcinoma (STAD) cohort of TCGA. The RNA‐sequencing data (log_2_ (TPM + 1) normalized) and matched DNA methylation microarray data (Illumina HumanMethylation450 BeadChip, represented by beta values ranging from 0 to 1) were downloaded via the UCSC Xena browser (https://xena.ucsc.edu/). To ensure strict cross‐omics alignment, samples were precisely matched using the first 15 characters of their TCGA barcodes. After excluding samples with missing expression or target probe methylation data, the paired dataset was extracted. Given the nonnormal distribution typical of tumor omics data, the nonparametric Spearman′s rank correlation test was utilized to evaluate the association between the beta values of prioritized CpG probes and corresponding mRNA expression levels. All statistical analyses and visualization (including scatter plots, fitted trend lines, and 95% confidence intervals) were performed in R (Version 4.3.3). A two‐tailed *p* − value < 0.05 was considered statistically significant.

### 2.8. Single‐Cell RNA Sequencing (scRNA‐seq) Analysis

To characterize the cell‐type‐specific distribution of prioritized candidate genes within the gastric TME, scRNA‐seq data (GSE270680) were retrieved from the GEO database. For each selected pathological subtype and stage category, one patient with paired tumor and adjacent normal samples was selected to evaluate tumor‐associated expression changes relative to the normal tissue baseline. In total, we selected 16 samples, including paired tumor and adjacent normal tissues from eight patients (Donors 02, 04, 08, 10, 12, 17, 22, and 24), encompassing distinct pathological subtypes (Stage II/III indeterminate, Stage I/II/III intestinal, and Stage I/II/III diffuse). The standard 10x Genomics files for these samples were downloaded and processed. Data processing was performed using the R package “Seurat” (v5.3.0). Stringent quality control criteria were applied to filter out low‐quality cells, retaining those meeting the following thresholds: (1) UMI counts between 500 and 50,000; (2) number of detected genes between 200 and 5000; and (3) mitochondrial gene proportion less than 25%. After putative doublets were manually removed, 114,794 high‐quality cells were retained for downstream analysis. The expression matrix was normalized using the NormalizeData function, and the top 2000 highly variable genes (HVGs) were identified using the FindVariableFeatures function with the vst method. Following data scaling using the ScaleData function, principal component analysis (PCA) was conducted using the RunPCA function. To reduce batch effects across different samples, batch correction was performed using the Harmony integration method implemented in the IntegrateLayers function in Seurat. Uniform Manifold Approximation and Projection (UMAP) was then utilized for nonlinear dimensionality reduction and visualization using the RunUMAP function based on the Top 20 principal components. Cells were clustered using the FindNeighbors and FindClusters functions based on a shared nearest‐neighbor graph constructed from the Top 20 principal components and subsequently annotated based on canonical cell‐type‐specific marker genes.

## 3. Results

### 3.1. ER Stress‐Related Gene Sets Retained Core Biological Characteristics After Filtering

To evaluate whether the filtering strategy preserved the biological features of the ER stress‐related gene set, GO and KEGG enrichment analyses were performed for the original gene set (2764 genes) and the final gene set (698 genes), with the latter used for SMR analysis. Both gene sets were significantly enriched in canonical ER stress‐related functional modules, including response to ER stress, response to topologically incorrect protein, response to unfolded protein, endoplasmic reticulum‐associated degradation (ERAD), and protein processing in the ER pathway (Tables S3 and S4; Figure S1A and S1B).

Compared with the original gene set, the final gene set showed a more focused enrichment pattern related to ER protein quality control and misfolded protein processing. These results suggest that the filtering and integration strategy retained the core biological characteristics of ER stress while yielding a more refined candidate gene set for subsequent SMR analysis.

### 3.2. ER Stress‐Related Gene Expression and GC Risk

The genetically supported associations between the genetically predicted expression levels of key ER stress‐related genes and the risk of GC are depicted in Figure 2A. In the FinnGen discovery cohort, a total of 10 ER stress‐related genes (*CTNNBIP1*, *PSMD4*, *ATXN3*, *RNF5*, *SELENOP*, *PPP1R12A*, *PCSK6*, *RETREG3*, *CEBPA*, and *UFD1*) showed genetic associations with GC risk, as detailed in Table S5. However, these associations did not reach statistical significance in the replication analyses using either the United Kingdom Biobank C16 or the United Kingdom Biobank 1018 GC cohorts, as shown in Tables S6 and S7. Among the initially identified signals, colocalization analysis provided strong evidence (PPH4 > 0.8) for one gene (*ATXN3*) and suggestive evidence (0.5 < PPH4 ≤ 0.8) for four genes (*CTNNBIP1*, *PSMD4*, *RETREG3*, and *UFD1)*. Specifically, the colocalization results for *CTNNBIP1* are illustrated in Figure 2A, with the regional distribution of the GC GWAS and eQTL signals for *CTNNBIP1* shown in Figure S3A.

**Figure 2:**
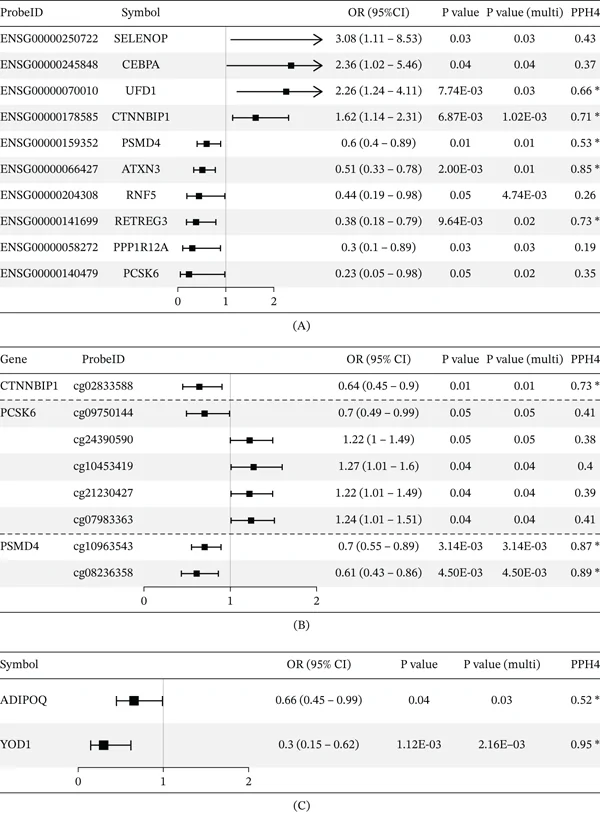
Forest plots of SMR analyses identifying key ER stress‐related molecular traits associated with gastric cancer risk. (A) Associations of genetically predicted key gene expression levels (eQTLs) with GC risk. (B) Associations of genetically predicted key DNA methylation levels at specific CpG sites (mQTLs) with GC risk. (C) Associations of genetically predicted key protein abundance (pQTLs) with GC risk. Only molecular traits passing the SMR significance thresholds are displayed. Error bars represent 95% confidence intervals.

### 3.3. ER Stress‐Related Gene Methylation and GC Risk

The complete results of the SMR analysis for ER stress‐related mQTL‐GWAS can be found in Table S8. A total of 83 CpG sites, corresponding to 53 genes, showed genetically supported associations with GC risk. Figure 2B shows the associations between selected CpG sites and GC risk for genes whose expression levels were also associated with GC risk. For instance, the CpG site cg24390590 located near the *PCSK6* gene was positively associated with the risk of GC (OR = 1.22, 95% CI = 1–1.49), whereas another CpG site within *PCSK6*, cg09750144, was negatively associated with GC risk (OR = 0.7, 95% CI = 0.49–0.99). Among the initially identified signals, colocalization evidence was observed for 47 sites corresponding to 33 genes. Of these, nine sites corresponding to eight genes met the threshold for strong colocalization evidence (PPH4 > 0.8), whereas the remaining sites showed suggestive evidence (0.5 < PPH4 ≤ 0.8). The colocalization result for cg02833588 corresponding to *CTNNBIP1* gene is shown in Figure S2B, with the regional distribution of the GC GWAS and mQTL signals for cg02833588 mapped to *CTNNBIP1* shown in Figure S3B.

Among the identified signals, the CpG site cg15982655 (annotated to *BNIP1*) was replicated in the United Kingdom Biobank C16 cohort, with the results detailed in Table S9. Additionally, the CpG sites cg04028464 and cg20269976 (annotated to *GAA*) were replicated in the United Kingdom Biobank 1018 GC cohort, with the corresponding findings presented in Table S10.

### 3.4. ER Stress‐Related Protein Abundance and GC Risk

The complete SMR analysis results for ER stress‐related pQTL‐GWAS are found in Table S11. Two ER stress‐related proteins, ADIPOQ and YOD1, were identified as negatively associated with GC risk, as illustrated in Figure 2C. ADIPOQ was also supported by suggestive colocalization evidence (0.5 < PPH4 ≤ 0.8), indicating a potential shared genetic basis with GC risk. The colocalization analysis provided strong evidence for YOD1 (PPH4 > 0.8), which is shown in Figure S2C. Nevertheless, these findings were not replicated in either the United Kingdom Biobank C16 or the United Kingdom Biobank 1018 GC cohorts, as reported in Tables S12 and S13.

### 3.5. Integrating Evidence From Multi‐Omics Levels

Based on the key results from the SMR analysis of mQTL‐GWAS, eQTL‐GWAS and pQTL‐GWAS, we further performed SMR analyses using mQTLs as the exposure and eQTLs as the outcome to investigate the genetic associations between DNA methylation and gene expression of ER stress‐related genes. In parallel, eQTLs were used as the exposure and pQTLs as the outcome to examine the genetic associations between gene expression and protein abundance of ER stress‐related genes across molecular layers. These analyses identified genetically supported associations between DNA methylation at key CpG sites and the expression of the corresponding genes, including cg02833588 in *CTNNBIP1*, cg09750144, cg24390590, cg10453419, cg21230427, and cg07983363 in *PCSK6*, as summarized in Table 1 and Table S14. The corresponding mQTL‐GWAS and eQTL‐GWAS signals for these prioritized genes are visualized as Manhattan plots in Figure 3. In contrast, no significant associations were observed between gene expression and protein abundance.

**Table 1:** Results of SMR analysis for the genetically supported associations between DNA methylation and gene expression.

Expo ID	Outco gene	*p* SMR	*p* SMR multi	*p* HEIDI	OR SMR (95% CI)
cg02833588	CTNNBIP1	7.57E‐14	7.57E‐14	0.174	0.4 (0.31–0.51)
cg09750144	PCSK6	3.98E‐06	3.98E‐06	0.933	1.24 (1.13–1.36)
cg24390590	PCSK6	2.67E‐09	2.67E‐09	0.357	0.87 (0.83–0.91)
cg10453419	PCSK6	6.27E‐09	6.27E‐09	0.143	0.85 (0.81–0.9)
cg21230427	PCSK6	1.90E‐09	1.90E‐09	0.235	0.87 (0.84–0.91)
cg07983363	PCSK6	4.28E‐08	4.28E‐08	0.551	0.88 (0.84–0.92)

**Figure 3:**
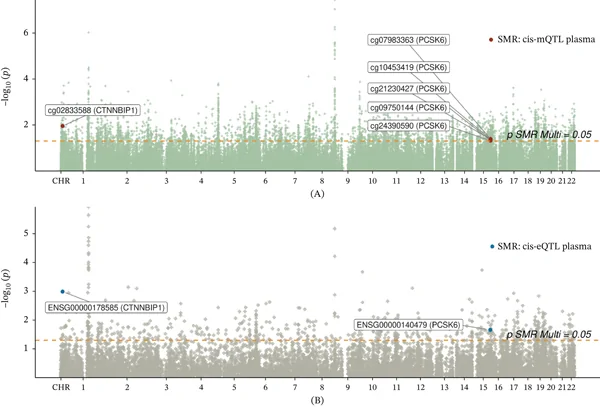
Manhattan plots of SMR analysis identifying ER stress‐related molecular traits associated with gastric cancer risk. (A) Manhattan plot displaying the association between DNA methylation sites (*cis*‐mQTL) and gastric cancer risk. Significant ER stress‐associated methylation probes for *CTNNBIP1* and a cluster of probes for *PCSK6* are highlighted. (B) Manhattan plot showing the association between gene expression levels (*cis*‐eQTL) and gastric cancer risk. Significant ER stress‐associated gene expression probes for *CTNNBIP1* and *PCSK6* are highlighted. The orange dashed line denotes the significance threshold (*P* − *S* *M* *R* − *m* *u* *l* *t* *i* = 0.05).

For *CTNNBIP1*, DNA methylation at the CpG site cg02833588 was negatively associated with GC risk, whereas *CTNNBIP1* expression was positively associated with GC risk. The mQTL‐eQTL analysis indicated that methylation at cg02833588 was negatively associated with *CTNNBIP1* expression. Together, these findings suggest that lower methylation at cg02833588 is genetically associated with higher *CTNNBIP1* expression, with both associated with higher GC risk.

Regarding *PCSK6*, methylation levels at different CpG sites showed distinct associations with GC risk. Specifically, methylation at cg09750144 was negatively associated with GC risk, whereas methylation at cg24390590, cg10453419, cg21230427, and cg07983363 were positively associated with GC risk. In contrast, *PCSK6* expression was negatively associated with GC risk. Moreover, mQTL‐eQTL analyses indicated that methylation at cg09750144 was positively associated with *PCSK6* expression, whereas methylation at cg24390590, cg10453419, cg21230427, and cg07983363 were negatively associated with its expression. Taken together, these results suggested that hypomethylation at cg09750144 and hypermethylation at cg24390590, cg10453419, cg21230427, and cg07983363 were associated with lower *PCSK6* expression and that both the methylation changes and lower *PCSK6* expression associated with higher GC risk. The regional distributions of the GC GWAS and mQTL signals for the selected *PCSK6* CpG sites, and of the GC GWAS and eQTL signals for *PCSK6*, are shown in Figure S4A and 4B. However, *PCSK6* did not show suggestive or strong colocalization evidence.

### 3.6. External Assessment in a GC Transcriptome Dataset

To further assess whether the expression patterns of SMR‐prioritized genes were directionally consistent with their genetically predicted associations with GC risk, we performed differential expression analysis using an independent GC gene expression dataset GSE118916. The complete results are presented in Table S15. Genes with *p* < 0.05 were considered differentially expressed. We then intersected these differentially expressed genes (DEGs) with SMR‐prioritized genes identified in the eQTL‐GWAS and pQTL‐GWAS analyses.

Among the six ER stress‐related genes overlapping between the SMR results and the DEGs identified in GSE118916 (*PSMD4*, *CTNNBIP1*, *PCSK6*, *YOD1*, *RNF5*, and *PPP1R12A*), three genes, namely *PCSK6*, *YOD1*, and *RNF5*, showed consistent expression changes in GSE118916 that aligned with the direction of their SMR associations. Specifically, *PCSK6* (log_2_ FC = −0.410, *p* = 0.00016), *YOD1* (log_2_ FC = −0.488, *p* = 0.00105), and *RNF5* (log_2_ FC = −0.355, *p* = 0.00116) were downregulated in GC tissues, consistent with the direction of their genetically predicted associations with GC risk. Notably, YOD1, identified through pQTL analysis, was additionally supported by strong colocalization evidence (PPH4 = 0.95). *PCSK6* and *RNF5* were identified through eQTL analysis. However, neither gene showed colocalization evidence at the predefined threshold. In contrast, *CTNNBIP1* (log_2_ FC = −0.340, *p* = 6.6 × 10^−5^), *PSMD4* (log_2_ FC = 0.618, *p* = 5.37 × 10^−12^), and *PPP1R12A* (log_2_ FC = 0.784, *p* = 0.003) exhibited expression patterns inconsistent with the direction of their genetically predicted associations. For instance, the SMR analysis suggested that increased *CTNNBIP1* expression was associated with a higher risk of GC, whereas transcriptomic analysis showed that *CTNNBIP1* was significantly downregulated in GC tissues. Boxplots of the differential expression results are shown in Figure 4, and the results are provided in Table S16.

**Figure 4:**
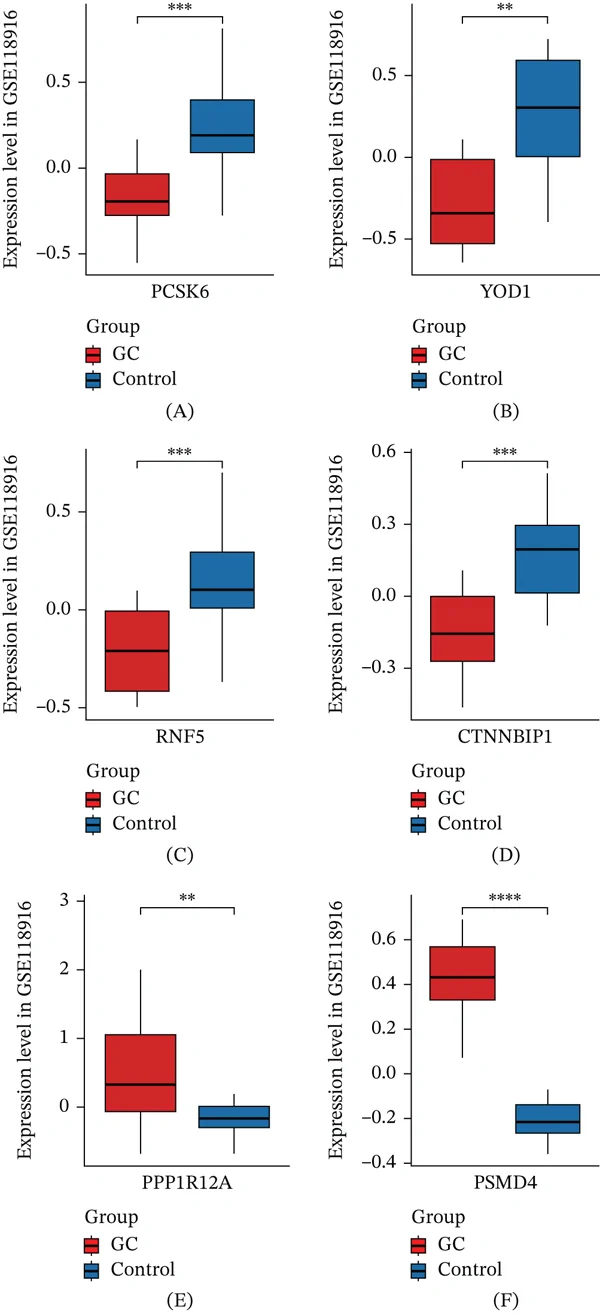
Boxplots showing differential expression of SMR‐prioritized candidate genes in the GSE118916 dataset. Expression levels in gastric tumor tissues (*n* = 15) compared with adjacent normal tissues (*n* = 15) for: (A) *PCSK6*, (B) *YOD1*, (C) *RNF5*, (D) *CTNNBIP1*, (E) *PPP1R12A*, and (F) *PSMD4*. Statistical comparisons were performed using the Wilcoxon rank‐sum test. Statistical significance was indicated by asterisks:  ^∗^ *p* < 0.05;  ^∗∗^ *p* < 0.01;  ^∗∗∗^ *p* < 0.001;  ^∗∗∗∗^ *p* < 0.0001.

### 3.7. Independent Methylation‐Expression Correlation Analysis in the TCGA‐STAD Cohort

To further evaluate the methylation‐expression associations suggested by our mQTL‐eQTL SMR analysis, we assessed the correlation between DNA methylation at prioritized CpG sites and target mRNA expression using the paired TCGA‐STAD cohort. The methylation level of cg02833588 exhibited a significant negative correlation with *CTNNBIP1* expression in GC tissues (Figure 5A). This observation was directionally consistent with the inverse methylation‐expression association suggested by the mQTL‐eQTL analysis.

**Figure 5:**
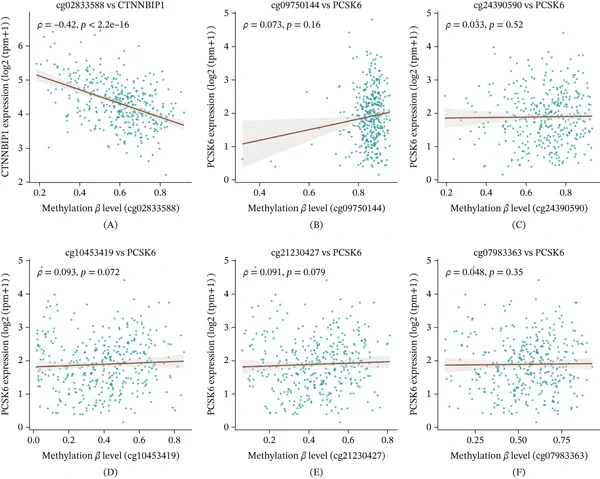
Correlation between DNA methylation and gene expression in the TCGA‐STAD cohort. Scatter plot showing the association between CpG site cg02833588 methylation level (beta value) and *CTNNBIP1* mRNA expression in paired gastric cancer samples (A) Scatter plots showing the associations between methylation levels of (B) cg09750144, (C) cg24390590, (D) cg10453419, (E) cg21230427, and (F) cg07983363 and *PCSK6* mRNA expression in paired gastric cancer samples. The fitted line represents the trend, and the shaded area indicates the 95% confidence interval. Spearman′s rank correlation coefficient (*ρ*) and *p*‐value are shown.

Regarding *PCSK6*, we did not observe statistically significant correlations between its mRNA expression and the methylation levels of the candidate CpG probes, including cg09750144, cg24390590, cg10453419, cg07983363, and cg21230427, in this cross‐sectional cohort (Figure 5B–F). It should be noted that although our SMR analysis captures the potential genetically supported associations based on germline genetic variants at the population level, TCGA methylation‐expression analysis reflects cross‐sectional associations within highly heterogeneous tumor tissues, where methylation effects may be context‐dependent. Therefore, these results should be interpreted as complementary cross‐sectional evidence that did not provide support for the genetically predicted methylation‐expression association of *PCSK6*. The potential relationship between *PCSK6* methylation and expression warrants further investigation in specific biological contexts.

### 3.8. Cell‐Type‐Specific Expression Patterns of Candidate Genes

To characterize the cell‐type‐specific expression of the prioritized genes (*PCSK6*, *YOD1*, and *RNF5*), we analyzed the scRNA‐seq dataset GSE270680. Before integration, PCA visualization showed partially dispersed distributions of cells from different samples in the low‐dimensional space (Figure S5A). Following Harmony integration, cells from different samples exhibited broader overlap and mixing in the integrated embedding, with less apparent sample‐specific separation (Figure S5B). Based on the expression profiles of canonical marker genes (e.g., *PTPRC*, *CD3D*, *EPCAM*, and *COL1A1*), we annotated 11 major cell types: CD4^+^ T cells, CD8^+^ T cells, innate lymphoid cells, gamma‐delta (*γδ*) T cells, B cells, plasma B cells, myeloid cells, epithelial cells, endothelial cells, fibroblasts, and mast cells (Figure 6A–C). Descriptive comparison between tumor and adjacent normal tissues showed distinct cell‐type‐specific expression patterns for the candidate genes (Figure 6D). *PCSK6* was predominantly expressed in fibroblasts. Notably, both the average expression level and the proportion of *PCSK6*‐expressing cells appeared higher in fibroblasts from adjacent normal tissues than in those from tumor tissues. *YOD1* was mainly expressed in epithelial cells and mast cells. Its average expression level and expressing‐cell proportion in epithelial cells appeared higher in adjacent normal tissues than in tumor tissues, whereas an opposite pattern was observed in mast cells, with higher average expression and a greater proportion of *YOD1*‐expressing cells in the tumor tissues. *RNF5* displayed a relatively broad cellular distribution, including mast cells, fibroblasts, epithelial cells, B cells, CD8^+^ T cells, and *γδ* T cells. The average expression levels of *RNF5* in fibroblasts and epithelial cells appeared relatively similar between tumor and adjacent normal tissues. Lower average expression levels were observed in mast cells, CD8^+^ T cells, and *γδ* T cells from tumor tissues, whereas higher average expression was observed in B cells from tumor tissues.

**Figure 6:**
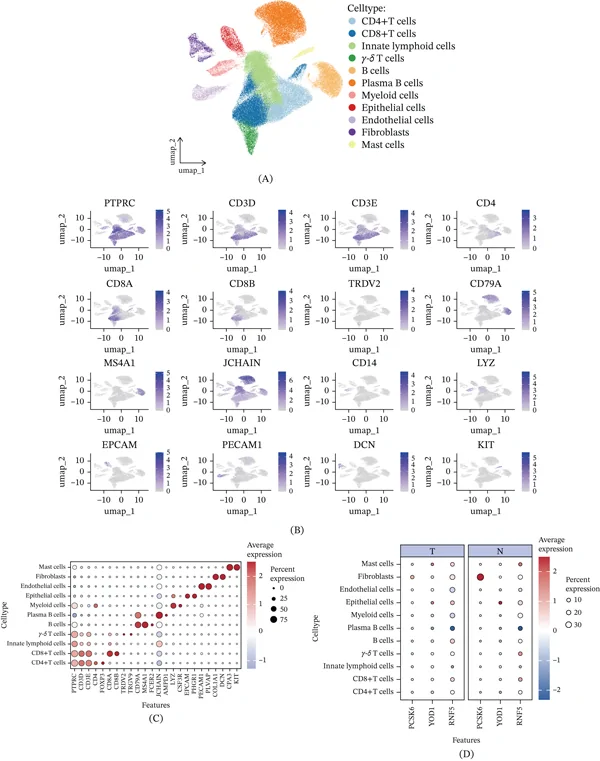
Single‐cell transcriptomic characterization of candidate gene expression in the gastric cancer microenvironment. (A) UMAP visualization of 11 annotated major cell types. (B) UMAP feature plots showing the expression of canonical marker genes used for cell‐type annotation. (C) Dot plot showing the expression patterns of representative cell‐type‐specific marker genes across the identified cell populations. (D) Expression patterns of *PCSK6*, *YOD1*, and *RNF5* in tumor (T) and adjacent normal (N) tissues across major cell types. Dot size represents the proportion of expressing cells, and color intensity represents the average expression level.

Collectively, these single‐cell transcriptomic analyses describe the cell‐type‐specific expression patterns of *PCSK6*, *YOD1*, and *RNF5* within the gastric tumor and adjacent normal tissues. These observations indicate that the prioritized candidate genes are detected not only in epithelial cells but also in stromal and immune cell populations. They therefore provide cellular context for the prioritized genes and should be regarded as hypothesis‐generating rather than as evidence of functional interactions, which would require dedicated functional experiments.

## 4. Discussion

In our study, we performed an exploratory multiomics SMR and colocalization analysis to systematically screen for genetic associations between ER stress‐related genes and GC risk. Our discovery analysis in the FinnGen cohort identified numerous candidate signals across DNA methylation, gene expression, and protein levels. By integrating multilayered external analyses, including transcriptomic profiling (GSE118916), methylation‐expression correlation analysis (TCGA‐STAD), and single‐cell transcriptomic characterization, we prioritized *PCSK6*, *YOD1*, and *RNF5* as ER stress‐related candidate genes associated with GC risk. These three genes showed differential expression patterns in tumor tissues that were directionally consistent with their SMR associations, providing additional context for their prioritization and subsequent functional investigation in GC. Among them, *YOD1* was supported by strong colocalization evidence, whereas *PCSK6* and *RNF5* did not show colocalization evidence at the predefined threshold but still showed directionally consistent transcriptomic patterns. Single‐cell analysis further described distinct cell‐type‐specific expression patterns: *PCSK6* was mainly expressed in fibroblasts, *YOD1* in epithelial cells and mast cells, and *RNF5* broadly across fibroblasts, epithelial cells, and selected immune cell populations, providing cellular context for these prioritized genes. Additionally, *CTNNBIP1* emerged as a noteworthy candidate. Although its differential expression pattern in GSE118916 was inconsistent with the SMR‐inferred direction, TCGA‐STAD analysis showed an inverse methylation‐expression correlation for cg02833588 and *CTNNBIP1*, which was directionally consistent with the mQTL‐eQTL association.

*PCSK6* is a member of the proprotein convertase family, which is involved in the activation of a variety of precursor proteins through proteolytic processing [27]. Altered *PCSK6* function has been linked to disturbances in protein processing and cellular homeostasis, providing a potential biological connection with ER stress‐related processes [28]. Given that ER stress is a well‐established contributor to cancer progression, altered *PCSK6* expression may affect protein homeostasis and ER stress‐related signaling in a context‐dependent manner. This may result in altered cellular signaling pathways relevant to cancer cell survival and metastatic behavior. *PCSK6* has been the subject of increasing attention in cancer research because of its potential roles in tumor progression and metastasis [29]. Our SMR analysis indicated an inverse association between the genetically predicted expression levels of the *PCSK6* gene and the risk of GC. The direction of this association was consistent with the lower *PCSK6* expression observed in GC tissues, although the underlying biological basis remains to be established. In the study by Delic et al., *PCSK6* was identified as a novel glioma invasion‐associated candidate gene, highlighting its potential role in the invasive phenotype of tumor cells [30]. Similarly, in pancreatic cancer, *PCSK6* was found to be crucial for liver metastasis, suggesting its involvement in the metastatic cascade [31]. These findings suggest that *PCSK6* may be involved in cellular processes related to tumor cell migration and metastasis. Furthermore, its role in breast cancer has been explored, where it was shown to activate the extracellular signal‐regulated kinase 1/2 (ERK1/2) and Wnt pathways, thereby promoting cancer cell proliferation, migration, and invasion [32]. These findings suggest that the function of *PCSK6* may vary across cancer types, cellular compartments, and tumor microenvironmental contexts. Mechanistically, previous studies have linked *PCSK6* to autophagy‐related processes [33]. Autophagy, a process that removes damaged cellular components, is essential for maintaining cellular homeostasis, especially in the context of stress. Altered *PCSK6* function may affect autophagy‐related processes in a context‐dependent manner, but its downstream consequences in cancer remain to be clarified. Additionally, *PCSK6* has been shown to modulate the activity of matrix metalloproteinases (MMPs), enzymes known for their role in extracellular matrix degradation and cell invasion, which are hallmarks of cancer metastasis [34]. The ability of *PCSK6* to regulate MMP activity, together with its reported associations with autophagy‐related processes and extracellular matrix remodeling, is noteworthy given our single‐cell finding that *PCSK6* is predominantly expressed in fibroblasts, with higher average expression and a greater proportion of expressing cells in adjacent normal fibroblasts than in fibroblasts from tumor tissues, whereas only relatively low expression was observed in epithelial cells in both groups. These findings may reflect tumor‐associated changes in *PCSK6* expression within the fibroblast compartment and provide a basis for further investigating its potential role in the GC microenvironment. Taken together, these findings provide biological context for the prioritization of *PCSK6* as an ER stress‐related candidate gene in GC. Nevertheless, these mechanistic interpretations are primarily based on previous literature and multiomics association evidence and therefore require further investigation through in vitro and in vivo functional experiments.

*YOD1*, with its deubiquitinase activity, is involved in the ERAD pathway, which is crucial for the quality control of proteins within the ER [35]. The ERAD pathway serves as a protective mechanism that helps maintain protein homeostasis by targeting misfolded proteins for degradation. Our study indicated that genetically predicted higher YOD1 protein abundance was associated with lower GC risk, raising the possibility that reduced YOD1 levels may be associated with impaired ERAD‐related protein quality control. Such alterations in protein quality control could influence cellular stress responses, although their downstream consequences in GC remain unclear. A transgenic mouse model demonstrated that the inducible expression of *YOD1* promotes hepatocyte proliferation and results in hepatomegaly in a YAP/TAZ‐activity‐dependent manner [36]. This finding underscores the context‐dependent effects of *YOD1* on cell proliferation and survival, potentially contributing to tumorigenesis. Furthermore, *YOD1* was found to be significantly overexpressed in triple‐negative breast cancer (TNBC) tissues compared to nontriple‐negative breast cancer (NTNBC) tissues [37]. This suggests that the role of *YOD1* may vary across tumor types and biological contexts. Bioinformatics analysis revealed that the Hippo signaling pathway, crucial for cell proliferation, survival, and apoptosis, is modulated by the cooperative action of *YOD1* and *USP21* [38, 39]. The Hippo pathway is known to regulate organ size, tissue homeostasis, and cancer progression, and its dysregulation is frequently observed in several types of cancer. The reported relationship between *YOD1* and the Hippo pathway raises the possibility that *YOD1* may be linked to cell survival and proliferation in GC through Hippo‐related signaling, although this possibility remains to be experimentally investigated. Notably, our single‐cell analysis revealed that *YOD1* was mainly expressed in epithelial cells and mast cells. In adjacent normal tissues, epithelial cells showed higher average *YOD1* expression and a greater proportion of *YOD1*‐expressing cells than those in tumor tissues, whereas mast cells showed the opposite pattern, with higher average expression and a greater expressing‐cell proportion in tumor tissues. This cell‐type‐specific pattern indicates variation in *YOD1* expression across cellular compartments, although its functional significance remains to be determined. In summary, the involvement of *YOD1* in ER‐associated protein quality control and ER stress‐related processes, together with its reported association with Hippo‐related signaling, provides biological context for its prioritization in our study and a basis for further functional and mechanistic investigation.

*RNF5*, a member of the RING finger protein family, has been implicated in various cellular processes, including the regulation of protein degradation through the ubiquitin‐proteasome system. *RNF5* is involved in the ERAD pathway, playing a critical role in targeting misfolded proteins for proteasomal degradation [40]. In our study, *RNF5* expression showed a direction of association similar to that of *PCSK6* and *YOD1*. Although the SMR association for *RNF5* was not supported by colocalization evidence in our study, the downregulation of *RNF5* in GC tissues suggests that lower *RNF5* expression may be associated with reduced efficiency of ER‐associated protein quality control, although whether this leads to misfolded protein accumulation or altered stress responses remains to be determined. Moreover, *RNF5* has been linked to the regulation of the NF‐*κ*B signaling pathway, a critical pathway involved in inflammation and cancer progression [41]. Given the established role of NF‐*κ*B in tumor‐associated inflammatory signaling, this relationship may provide a potential mechanistic context for investigating *RNF5* in GC, although its specific relevance remains unclear. Our single‐cell analysis further revealed that *RNF5* was not restricted to epithelial cells but was broadly distributed across fibroblasts and multiple immune cell populations. Notably, average *RNF5* expression was higher in mast cells, CD8^+^ T cells, and *γδ* T cells from adjacent normal tissues than in the corresponding cell types from tumor tissues. Given that CD8^+^ T cells and *γδ* T cells are important antitumor effector cells, the lower *RNF5* expression observed in these populations may warrant further investigation regarding its potential relevance to local antitumor immune responses. Meanwhile, decreased *RNF5* expression in mast cells may be relevant to tumor‐associated inflammatory processes, although this possibility requires further investigation. Conversely, average *RNF5* expression was higher in B cells from tumor tissues than in adjacent normal B cells, suggesting a distinct cell‐type‐specific expression pattern. B cells can exert antitumor functions, but certain immunosuppressive B‐cell subsets may also favor tumor progression. The biological significance of the higher *RNF5* expression observed in B cells from tumor tissues therefore remains to be clarified. Together, these single‐cell findings highlight the broad and cell‐type‐specific expression patterns of *RNF5* across multiple cellular compartments, providing a basis for further investigating its potential role in the gastric TME. Overall, our genetic, transcriptomic, and single‐cell findings provide complementary evidence supporting the prioritization of *RNF5* as an ER stress‐related candidate gene in GC. Its established involvement in ER‐associated protein quality control provides additional biological context for this prioritization. Its broad and heterogeneous expression across epithelial, stromal, and immune compartments also suggests that its biological relevance may vary across cellular contexts. Further functional studies are required to clarify its precise role in GC.

In addition to the above three genes, *CTNNBIP1* represents an interesting case. Its genetically predicted expression was positively associated with GC risk in the SMR analysis, with additional genetic evidence from mQTL and mQTL‐eQTL analyses, as well as a significant negative methylation‐expression correlation in the TCGA‐STAD cohort. However, *CTNNBIP1* was downregulated in tumor tissues in the GSE118916 dataset, a pattern that did not match the direction of its genetically predicted association. This apparent discrepancy should be interpreted with caution, because the two types of evidence reflect different biological layers. Genetically predicted molecular effects derived from germline variants mainly reflect the influence of inherited genetic background on a molecular trait at the population level, whereas bulk tumor transcriptomic data such as GEO and TCGA reflect the combined outcome of multiple factors operating in the diseased tissue, including the TME, changes in cell composition, disease stage, and other acquired regulatory events. An incomplete agreement between the genetically predicted direction and the observed tissue expression change therefore does not necessarily indicate a contradiction, and may instead reflect context‐dependent differences between genetically predicted molecular effects and observed tumor‐tissue expression. Given this divergence, the biological relevance of *CTNNBIP1* in GC requires further functional investigation.

More broadly, direct GC‐specific functional evidence for these prioritized genes remains limited, particularly regarding their involvement in ER stress. Existing GC‐related studies have implicated *PCSK6* in macrophage polarization [42], *RNF5* in ubiquitination‐dependent regulation of autophagy [43, 44], and *CTNNBIP1* in Wnt/*β*‐catenin‐related processes [45–47], whereas direct functional evidence for *YOD1* in GC remains sparse. Importantly, these studies do not establish that these genes affect GC development through ER stress‐related mechanisms. Accordingly, the mechanistic interpretations presented above should be considered hypothesis‐generating and interpreted in the context of known gene functions together with our multiomics and single‐cell findings. Further pathway‐focused analyses and functional studies in GC models are required to clarify whether and how these genes are involved in ER stress‐related processes, TME‐associated changes, and GC progression.

To fully appreciate the potential roles of these ER stress‐related genes, it is essential to consider their possible spatial context within the gastric TME. Although our current study is rooted in genetic association and gene prioritization, recent high‐resolution spatial transcriptomic studies provide valuable biological background support [48]. These studies map the spatial architecture of GC, highlighting how cellular spatial distribution varies across different regions, particularly noting that lymphocyte aggregated regions and tertiary lymphoid structures (TLSs) serve as focal points for antitumor immunity. In these structured regions, activated lymphocytes and specific dendritic cells cluster in adjacent cancerous zones to modulate immune responses. Future integration of our prioritized candidate genes with spatial transcriptomic and cell–cell communication analyses may help clarify whether their expression and associated molecular patterns exhibit spatial differences across tumor‐stromal and immune niches, offering a direction for future microenvironmental research.

A strength of this research is its comprehensive and systematic assessment of genetically regulated molecular traits related to ER stress and GC risk. A notable strength of our study lies in the integrative framework that combines multiomics data (mQTL, eQTL, and pQTL) with multilayered external analyses, including transcriptomic profiling, methylation‐expression correlation analysis, and single‐cell transcriptomic characterization. This integrative approach enabled us to systematically narrow down a broad ER stress‐related gene set to a smaller set of prioritized candidates. Rather than serving as an endpoint, our multiomics investigation provides a prioritized list of ER stress‐related genes associated with GC risk for subsequent functional investigation. Taken together with the cell‐type‐specific expression patterns observed here, these findings highlight the heterogeneous cellular distribution of the prioritized genes across epithelial, stromal, and immune compartments. However, these expression patterns do not establish functional interactions among these cellular compartments, and dedicated functional and spatial studies will be needed to investigate this possibility.

However, several limitations must be acknowledged. First, it is important to recognize that SMR analysis is inherently a statistical inference tool based on genetic evidence. Consequently, our findings primarily serve the purpose of gene prioritization rather than providing definitive causal proof. Second, although our analytical framework, incorporating the HEIDI test and Bayesian colocalization, helps evaluate and reduce the possibility of spurious associations driven by LD without fully eliminating this possibility, residual horizontal pleiotropy remains an inherent limitation of all MR studies. Moreover, although PPH4 > 0.5 was used as the threshold for exploratory screening of potential shared genetic signals, this relatively lenient threshold introduces greater uncertainty than more stringent criteria. Accordingly, suggestive colocalization evidence should be interpreted in combination with SMR results, HEIDI testing, multiomics consistency, and external supporting evidence. Third, although the external replication analyses were conducted in the independent United Kingdom Biobank cohorts, several associations identified in the FinnGen discovery cohort were not consistently replicated. Several factors may contribute to this discrepancy, and they are not mutually exclusive: the smaller number of GC cases in the United Kingdom Biobank cohorts may have limited the statistical power to detect small‐to‐moderate genetic effects, whereas differences in population characteristics, genetic background, and phenotype definitions may also have contributed to between‐population heterogeneity. We therefore do not attribute the incomplete replication to any single cause, and nonreplication should not be interpreted as conclusive evidence against these associations. Accordingly, these findings should be interpreted as genetically supported prioritization of candidate genes rather than definitive identification of causal genes, and priority was given to candidate genes supported by multiple lines of evidence, including SMR analysis, HEIDI testing, colocalization analysis, transcriptomic analysis, TCGA methylation‐expression correlation analysis, and single‐cell expression patterns. Future studies in larger, independent GC GWAS cohorts with more consistent phenotype definitions, together with functional experiments, are needed to further evaluate and replicate these associations and explore the underlying biological mechanisms. Fourth, although large‐scale blood‐based QTL data provided improved statistical power, the current lack of analysis using large‐scale stomach‐specific eQTL data limits our ability to fully capture tissue‐contextual effects. Future availability of stomach‐specific QTL datasets may provide additional evidence regarding the tissue context of these associations. Fifth, regarding data sources, although a larger data release (FinnGen R12) has recently become available, our study was conducted utilizing the R10 release. Sixth, the candidate gene set was constructed by integrating GeneCards‐derived ER stress‐related genes with two MSigDB gene sets. Different databases and curated gene sets may partially overlap without fully reflecting the same biological aspects of ER stress. Although functional enrichment analysis supported the preservation of core ER stress‐related biological functions, differences in database curation and annotation strategies may still affect the composition of the candidate gene set, and prioritization based on GeneCards relevance scores may introduce database‐dependent bias. Incorporating more experimentally validated ER stress‐related gene signatures in future studies may help refine the candidate gene landscape. Seventh, although we utilized single‐cell transcriptomic data to characterize the cell‐type‐specific expression patterns of the candidate genes, the specific functional roles of these genes in different cell types cannot be determined based solely on expression data. Furthermore, we did not integrate spatial transcriptomic or cell–cell communication analyses to assess their spatial distribution patterns and potential intercellular interactions within the TME. Finally, direct functional evidence linking these ER stress‐related candidate genes to GC remains scarce. The available studies provide useful biological clues, but they do not yet demonstrate that these genes contribute to gastric tumorigenesis through ER stress or ERAD‐related pathways. Thus, the mechanistic interpretations proposed here should be viewed as working hypotheses rather than established biological mechanisms. Further studies using pathway‐level analyses and experimental GC models are warranted to clarify whether and how these genes are involved in ER stress‐related processes, TME‐associated changes, and disease progression.

## 5. Conclusions

In conclusion, the present study used SMR analysis to investigate the genetically supported associations between DNA methylation, gene expression, protein abundance, and GC risk in the context of ER stress‐related biology. This work prioritized ER stress‐related candidate genes, including *PCSK6*, *YOD1*, and *RNF5*, and provided preliminary genetic evidence for their association with GC risk. Single‐cell transcriptomic analysis further revealed distinct cell‐type‐specific expression patterns of these candidates, providing cellular context for the prioritized genes. These findings are hypothesis‐generating and provide a basis for further investigation into the biological significance of these candidate genes across different cellular contexts. Although the evidence provided is exploratory, additional functional and mechanistic studies are required to better understand their potential roles in GC.

**Nomenclature**
BH: Benjamini–Hochberg
CI: confidence interval
DEG: differentially expressed gene
EBV: Epstein–Barr virus
ER: endoplasmic reticulum
ERAD: endoplasmic reticulum‐associated degradation
ERK: extracellular signal‐regulated kinase
eQTL: expression quantitative trait locus
GC: gastric cancer
GEO: Gene Expression Omnibus
GO: Gene Ontology
GTEx: Genotype‐Tissue Expression
GWAS: genome‐wide association study
HEIDI: heterogeneity in dependent instruments
HVG: highly variable gene
KEGG: Kyoto Encyclopedia of Genes and Genomes
LD: linkage disequilibrium
MMP: matrix metalloproteinase
MR: Mendelian randomization
mQTL: methylation quantitative trait locus
NTNBC: nontriple‐negative breast cancer
OR: odds ratio
PCA: principal component analysis
PPH4: posterior probability for hypothesis 4
pQTL: protein quantitative trait locus
QTL: quantitative trait locus
scRNA‐seq: single‐cell RNA sequencing
SMR: summary‐data‐based Mendelian randomization
STAD: stomach adenocarcinoma
TAZ: transcriptional coactivator with PDZ‐binding motif
TCGA: The Cancer Genome Atlas
TLS: tertiary lymphoid structure
TME: tumor microenvironment
TNBC: triple‐negative breast cancer
TNM: tumor‐node‐metastasis
UKB: UK Biobank
UMI: unique molecular identifier
UMAP: Uniform Manifold Approximation and Projection
UPR: unfolded protein response
USP21: ubiquitin‐specific protease 21
YAP: Yes‐associated protein

## Author Contributions

Conception and design: Dong Yang, Yuanlin Liu, and Tao Zhang. Administrative support: Xiangyu Meng. Provision of study materials or patients: Chao Wang and Zhiyong Yu. Collection and assembly of data: Dong Yang and Yuanlin Liu. Data analysis and interpretation: Dong Yang and Yuanlin Liu. Manuscript writing: Dong Yang, Yuanlin Liu, Xiangyu Meng, Chao Wang, Zhiyong Yu, and Tao Zhang. Final approval of manuscript: Dong Yang, Yuanlin Liu, Xiangyu Meng, Chao Wang, Zhiyong Yu, and Tao Zhang.

## Funding

This study was supported by the Joint Program of Science and Technology Program of Liaoning Province (Natural Science Foundation‐General Program), 2024‐MSLH‐274; and the Wu Jieping Medical Fund, 320.6750.2024‐25‐13.

## Ethics Statement

The authors have nothing to report.

## Consent

The authors have nothing to report.

## Conflicts of Interest

The authors declare no conflicts of interest.

## Supporting Information

Additional supporting information can be found online in the Supporting Information section.

## Supporting information


**Supporting Information 1** Figure S1: Functional enrichment analysis of ER stress‐related gene sets. (A) GO and KEGG enrichment results for the original gene set (2764 genes). (B) GO and KEGG enrichment results for the final gene set used for SMR analysis (698 genes). Enrichment *p* values were adjusted using the Benjamini–Hochberg method, and terms with adjusted *p* < 0.05 were considered significantly enriched. Dot size represents the number of genes enriched in the corresponding term, and colors denote the functional categories.

**Supporting Information 2** Figure S2: Colocalization plots for ER stress‐related molecular traits and GC risk. (A) Colocalization plot for *CTNNBIP1* expression and GC risk. (B) Colocalization plot for DNA methylation at CpG site cg02833588 (annotated to *CTNNBIP1*) and GC risk. (C) Colocalization plot for YOD1 protein abundance and GC risk.

**Supporting Information 3** Figure S3: Locus‐specific SMR and QTL signals for *CTNNBIP1* associated with GC risk. (A) Integrative plot for gene expression, showing the regional distribution of GC GWAS/SMR signals (top) with *CTNNBIP1 cis*‐eQTL signals (middle) and the genomic gene track (bottom). (B) Integrative plot for DNA methylation, showing the regional distribution of GC GWAS/SMR signals (top) with *CTNNBIP1 cis*‐mQTL signals at probe cg02833588 (middle) and the genomic gene track (bottom). The red horizontal dashed line represents the significance threshold (*P-SMR-multi* = 0.05). Gold arrows indicate the position and orientation of *CTNNBIP1* and neighboring genes in the region.

**Supporting Information 4** Figure S4: Locus‐specific SMR and QTL signals for *PCSK6* associated with GC risk. (A) Integrative plot for DNA methylation, showing the regional distribution of GC GWAS/SMR signals (top) with *PCSK6 cis*‐mQTL signals for multiple significant probes (middle) and the genomic gene track (bottom). (B) Integrative plot for gene expression, showing the regional distribution of GC GWAS/SMR signals (top) with the *PCSK6 cis*‐eQTL signal (middle) and the genomic gene track (bottom). The red horizontal dashed line represents the significance threshold (*P-SMR-multi* = 0.05). Gold arrows indicate the position and orientation of *PCSK6* and neighboring genes in the region.

**Supporting Information 5** Figure S5: Low‐dimensional visualization of single‐cell data before and after Harmony integration. (A) PCA visualization of cells from different samples before Harmony integration. (B) Harmony embedding of cells from different samples after integration. Colors represent different sample origins.

**Supporting Information 6** Table S1: Information on included studies and consortia. Table S2: Summary of ER stress‐related gene sets derived from GeneCards and MSigDB for SMR analysis. Table S3: GO enrichment and KEGG pathway enrichment results for the original ER stress‐related gene set. Table S4: GO enrichment and KEGG pathway enrichment results for the final ER stress‐related gene set used for SMR analysis. Table S5: SMR analysis results for endoplasmic reticulum stress‐related eQTL‐GWAS in the FinnGen cohort. Table S6: SMR analysis results for endoplasmic reticulum stress‐related eQTL‐GWAS in the UKB C16 cohort. Table S7: SMR analysis results for endoplasmic reticulum stress‐related eQTL‐GWAS in the UKB 1018 GC cohort. Table S8: SMR analysis results for endoplasmic reticulum stress‐related mQTL‐GWAS in the FinnGen cohort. Table S9: SMR analysis results for endoplasmic reticulum stress‐related mQTL‐GWAS in the UKB C16 cohort. Table S10: SMR analysis results for endoplasmic reticulum stress‐related mQTL‐GWAS in the UKB 1018 GC cohort. Table S11: SMR analysis results for endoplasmic reticulum stress‐related pQTL‐GWAS in the FinnGen cohort. Table S12: SMR analysis results for endoplasmic reticulum stress‐related pQTL‐GWAS in the UKB C16 cohort. Table S13: SMR analysis results for endoplasmic reticulum stress‐related pQTL‐GWAS in the UKB 1018 GC cohort. Table S14: SMR analysis results for mQTL‐eQTL associations. Table S15: Differentially expressed genes in the GSE118916 dataset. Table S16: Differential expression analysis of SMR‐prioritized genes in the independent GC dataset GSE118916.

**Supporting Information 7** File S1: STROBE‐MR checklist of recommended items to address in reports of Mendelian randomization studies.

## Data Availability

All data analyzed in this study were obtained from publicly available databases, and the relevant results are included in this article and its supporting information.
