# Supplementary material for: Integrative Multiomics Mendelian Randomization Prioritizes Endoplasmic Reticulum Stress‐Related Genes Associated With Gastric Cancer Risk
Source: Hum Mutat. 2026 Oct 27;2026:3845963. doi: 10.1155/humu/3845963 (PMC13714348; doi:10.1155/humu/3845963)
Supplement: Supplementary file 1 [file HUMU-2026-3845963-s007.docx]

**STROBE-MR checklist of recommended items to address in reports of Mendelian randomization studies**^1^ ^2^

| **Item No.** | **Section** | **Checklist item** | **Page No.** | **Relevant text from manuscript** |
| --- | --- | --- | --- | --- |
| 1 | **TITLE and ABSTRACT** | Indicate Mendelian randomization (MR) as the study’s design in the title and/or the abstract if that is a main purpose of the study | 1–3 | Title (p. 1).  "Integrative Multi-omics Mendelian Randomization Prioritizes Endoplasmic Reticulum Stress-Related Genes Associated with Gastric Cancer Risk" (p. 1).  Running title (p. 1).  "Multi-omics MR of ER Stress in Gastric Cancer” (p. 1).  Abstract, Methods (p. 2).  "Summary-data-based Mendelian randomization (SMR) and colocalization analyses were performed to prioritize candidate genes, followed by characterization using transcriptomic profiling (GSE118916), methylation-expression correlation analysis (TCGA-STAD), and single-cell RNA-seq analysis (GSE270680)." (p. 2).  Abstract, Results (p. 2).  "SMR analyses identified 83 mQTL, 10 eQTL, and 2 pQTL signals associated with GC risk..." (p. 2).  Keywords (p. 3).  "Gastric cancer; Endoplasmic reticulum stress; Multi-omics; Mendelian randomization analysis; Colocalization; Single-cell RNA sequencing" (p. 3). |
|  | **INTRODUCTION** |  |  |  |
| 2 | **Background** | Explain the scientific background and rationale for the reported study. What is the exposure? Is a potential causal relationship between exposure and outcome plausible? Justify why MR is a helpful method to address the study question | 4–6 | Introduction (p. 4).  "Gastric cancer (GC) is one of the most prevalent malignancies worldwide and remains the fourth leading cause of cancer-related mortality [1]…a deeper understanding of the genetic and molecular factors associated with GC development…" (p. 4).  "Accumulating evidence indicates that genetic alterations and dysregulated molecular pathways play important roles in GC biology... endoplasmic reticulum (ER) stress and the unfolded protein response (UPR) have been implicated in several malignant phenotypes of GC..." (pp. 4–5).  "However, many existing studies of ER stress in GC have relied on observational designs or small-scale transcriptomic profiling, which may be affected by reverse causality, confounding factors, and limited reproducibility… Consequently, the genetic factors and molecular networks specifically linking ER stress to GC risk have not been systematically characterized…" (p. 5).  "Summary-data-based Mendelian randomization (SMR) provides a genetic framework for integrating publicly available genome-wide association study (GWAS) summary statistics with quantitative trait loci (QTL) data for DNA methylation, gene expression, and protein abundance to prioritize genes whose genetically regulated molecular features may be associated with disease phenotypes [8]... In cancer research, including GC, the integration of SMR, Heterogeneity in Dependent Instruments (HEIDI) testing, and colocalization has provided a useful strategy for prioritizing genetically supported candidate genes and molecular traits across different cancer types and omics layers [11-13]…" (pp. 5–6). |
| 3 | **Objectives** | State specific objectives clearly, including pre-specified causal hypotheses (if any). State that MR is a method that, under specific assumptions, intends to estimate causal effects | 6 | Introduction (p. 6).  "In this study, the combined use of SMR, HEIDI, and colocalization analysis provided a framework for prioritizing ER stress-related genes whose genetically regulated molecular traits were associated with GC risk, while distinguishing shared genetic signals from associations attributable to LD. By integrating SMR analysis, Gene Expression Omnibus (GEO) transcriptomic profiling, The Cancer Genome Atlas (TCGA) methylation-expression correlation analysis, and single-cell transcriptomic data, this study systematically evaluated ER stress-related candidate genes across multiple dimensions, including genetic associations, methylation-expression correlations, transcriptional alterations, and cell-type-specific expression patterns. Our findings provide supporting evidence for the prioritization of candidate genes for subsequent functional and mechanistic investigations." (p. 6).  Additional note: No specific causal hypothesis was pre-specified in the present study. The study was designed primarily to prioritize ER stress-related candidate genes with genetically supported associations with GC risk using the SMR framework, rather than to establish definitive causal effects. |
|  | **METHODS** |  |  |  |
| 4 | **Study design and data sources** | Present key elements of the study design early in the article. Consider including a table listing sources of data for all phases of the study. For each data source contributing to the analysis, describe the following: | 6–8 | Materials and Methods, Study Design. (pp. 6–7).  "In our study, we identified instrumental variables associated with ER stress-related molecular traits across three omics layers: DNA methylation, gene expression and protein abundance. To explore their potential genetic associations with GC risk, we conducted SMR analyses at each molecular layer. The primary dataset for discovery was derived from the FinnGen R10 cohort, and for the replication dataset, we utilized GC GWAS data from the UK Biobank cohorts…By integrating multi-omics SMR analysis with HEIDI and colocalization assessment, we prioritized ER stress-related candidate genes with genetic evidence of association with GC risk and subsequently performed further characterization using GEO transcriptomic expression profiles, TCGA methylation-expression correlation analysis, and single-cell transcriptomic data." (pp. 6–7).  "Figure 1 presents an overview of the study framework and the main analytical procedures." (p. 7).  Materials and Methods, Data Sources (pp. 7–8).  "Detailed information on all GWAS datasets used in this study is summarized in Supplementary Table S1." (p. 7).  "The original GeneCards-derived genes, the high-priority GeneCards candidates, and the final integrated ER stress-related gene set are provided in Supplementary Table S2." (p. 8).  "Detailed information on all QTL datasets used in this study is summarized in Supplementary Table S1." (p. 8). |
|  | a) | Setting: Describe the study design and the underlying population, if possible. Describe the setting, locations, and relevant dates, including periods of recruitment, exposure, follow-up, and data collection, when available. | 7 | Materials and Methods, Data Sources (p. 7).  "We obtained the GWAS summary statistics for GC from the FinnGen R10 cohort (https://storage.googleapis.com/finngen-public-data-r10/summary_stats/finngen_R10_C3_STOMACH_EXALLC.gz), including 1,423 cases and 314,193 controls... For replication, we used two independently defined GC phenotypes from the UK Biobank. The first was an ICD-10-based phenotype for malignant neoplasm of the stomach (C16), comprising 764 cases and 419,767 controls. The second was based on the UK Biobank self-reported cancer code 1018 (stomach cancer), comprising 155 cases and 420,318 controls... Detailed information on all GWAS datasets used in this study is summarized in Supplementary Table S1." (p. 7).  Additional note: As this study relied on publicly available summary-level datasets, detailed information on recruitment periods, exposure periods, follow-up periods, and data collection dates was not described in the present manuscript. |
|  | b) | Participants: Give the eligibility criteria, and the sources and methods of selection of participants. Report the sample size, and whether any power or sample size calculations were carried out prior to the main analysis | 7–8 | Materials and Methods, Data Sources (pp. 7–8).  "We obtained the GWAS summary statistics for GC from the FinnGen R10 cohort (https://storage.googleapis.com/finngen-public-data-r10/summary_stats/finngen_R10_C3_STOMACH_EXALLC.gz), including 1,423 cases and 314,193 controls... For replication, we used two independently defined GC phenotypes from the UK Biobank. The first was an ICD-10-based phenotype for malignant neoplasm of the stomach (C16), comprising 764 cases and 419,767 controls. The second was based on the UK Biobank self-reported cancer code 1018 (stomach cancer), comprising 155 cases and 420,318 controls... Detailed information on all GWAS datasets used in this study is summarized in Supplementary Table S1." (p. 7).  "We acquired blood eQTL data from the eQTLGen Consortium, encompassing gene expression profiles for 31,684 individuals [16]. The mQTL data were obtained through a meta-analysis of two European cohorts: the Brisbane Systems Genetics Study, which involved 614 participants, and the Lothian Birth Cohorts, which included 1,366 participants [17]. Additionally, pQTL data were retrieved from a study by Sun BB et al., which comprised 54,219 participants from the UK Biobank [18]. Detailed information on all QTL datasets used in this study is summarized in Supplementary Table S1." (p. 8).  Additional note: As this study was a secondary analysis based on publicly available GWAS and QTL summary statistics, detailed individual-level eligibility criteria and participant selection procedures were not described in the present manuscript, and no formal a priori power or sample size calculation was performed for the present analysis. |
|  | c) | Describe measurement, quality control and selection of genetic variants | 8–10 | Materials and Methods, Summary-data-based MR Analysis (pp. 8–10).  "Summary-data-based Mendelian randomization (SMR) was performed to evaluate the potential genetically supported associations between genetically regulated molecular traits of ER stress-related genes... and GC risk. Within the SMR framework, the three core assumptions of MR were carefully considered. First, for the relevance assumption, genome-wide significant cis-QTLs located within ±1 Mb of the target gene were selected as instrumental variables... Second... only cis-acting variants near the target gene were used... Third, for the exclusion restriction assumption, SMR does not rely on the conventional stringent linkage disequilibrium (LD) clumping strategy commonly used in multi-instrument MR. Instead, it leverages the local LD structure within the cis-region and evaluates potential LD-driven associations using the HEIDI test and, where applicable, the SMR-multi extension." (pp. 8–9).  "The selection of top cis-QTLs was based on a genomic window spanning ±1000 kb around the gene of interest, coupled with a stringent significance threshold of P < 5.0 × 10^-8^ [20, 21]. In the standard single-SNP SMR analysis, the top genome-wide significant cis-QTL within ±1 Mb of the target gene was selected as the primary instrumental variable... SNPs with allele frequency differences greater than 0.2 across the LD reference panel, QTL datasets, and outcome GWAS datasets were excluded to avoid potential bias from population structure differences." (p. 9).  "Building upon the standard SMR framework, we further utilized a multi-SNP-based SMR approach (--smr-multi)... all SNPs within a specific QTL probe window (default ±500 kb) that satisfy two criteria: P < 5.0 × 10^-8^ and linkage disequilibrium r^2^ < 0.9 with the top associated SNP [22]." (p. 10). |
|  | d) | For each exposure, outcome, and other relevant variables, describe methods of assessment and diagnostic criteria for diseases | 7–8 | Materials and Methods, Data Sources (pp. 7–8).  "GC cases in the FinnGen cohort were defined using registry-based diagnostic codes, including ICD-10 code C16 and the corresponding ICD-8/9 codes, together with ICD-O-3 topography code C16 from the Finnish Cancer Registry… For replication, we used two independently defined GC phenotypes from the UK Biobank. The first was an ICD-10-based phenotype for malignant neoplasm of the stomach (C16)… The second was based on the UK Biobank self-reported cancer code 1018 (stomach cancer)…" (p. 7).  "Using the keyword 'endoplasmic reticulum stress', we retrieved 2,764 genes from the GeneCards database... After restricting the results to protein-coding genes and ranking them by relevance score, we retained the top 25% of protein-coding genes, corresponding to 586 candidate genes... we incorporated two ER stress-related gene sets from the Molecular Signatures Database (MSigDB)... The union of these sources yielded a final set of 698 ER stress-related genes for subsequent SMR analysis." (p. 8). |
|  | e) | Provide details of ethics committee approval and participant informed consent, if relevant | 7, 35 | Materials and Methods, Study Design. (p. 7).  "All summary statistics used in the SMR analysis were obtained from previously published studies or publicly available resources with appropriate ethical approval." (p. 7).  Declarations, Ethics approval and consent to participate (p. 35).  "Not applicable." (p. 35). |
| 5 | **Assumptions** | Explicitly state the three core IV assumptions for the main analysis (relevance, independence and exclusion restriction) as well assumptions for any additional or sensitivity analysis | 8–11 | Materials and Methods, Summary-data-based MR Analysis (pp. 8–10).  "Within the SMR framework, the three core assumptions of MR were carefully considered. First, for the relevance assumption, genome-wide significant cis-QTLs located within ±1 Mb of the target gene were selected as instrumental variables... Second, for the independence assumption, only cis-acting variants near the target gene were used... Third, for the exclusion restriction assumption, SMR does not rely on the conventional stringent linkage disequilibrium (LD) clumping strategy commonly used in multi-instrument MR. Instead, it leverages the local LD structure within the cis-region and evaluates potential LD-driven associations using the HEIDI test and, where applicable, the SMR-multi extension." (pp. 8–9).  "This approach enhanced statistical power when using top cis-QTLs compared with the conventional MR analysis [19]." (p. 9).  "Besides exploring the potential genetically supported associations between molecular QTLs (mQTLs, eQTLs, and pQTLs) and GC risk, we further explored cross-omics genetic associations, treating mQTLs as exposures and eQTLs as outcomes, as well as eQTLs as exposures and pQTLs as outcomes." (pp. 9–10).  "Building upon the standard SMR framework, we further utilized a multi-SNP-based SMR approach (--smr-multi)…" (p. 10).  "Subsequently, the HEIDI test was applied to assess heterogeneity potentially attributable to linkage disequilibrium and to help distinguish shared genetic signals from associations driven by distinct linked variants." (p. 10).  Materials and Methods, Colocalization Analysis (pp. 10–11).  "Colocalization analysis was used to identify whether ER stress-related cis-QTLs (including eQTLs, mQTLs, and pQTLs) and GC GWAS signals were likely to share a common causal variant…" (pp. 10–11). |
| 6 | **Statistical methods: main analysis** | Describe statistical methods and statistics used |  |  |
|  | a) | Describe how quantitative variables were handled in the analyses (i.e., scale, units, model) | – | Additional note: For the binary GC outcome, effect estimates were presented as odds ratios (ORs) with 95% confidence intervals. The specific measurement scales and units of the molecular traits were not explicitly described in the present manuscript. |
|  | b) | Describe how genetic variants were handled in the analyses and, if applicable, how their weights were selected | 9–10 | Materials and Methods, Summary-data-based MR Analysis (pp. 9–10).  "The selection of top cis-QTLs was based on a genomic window spanning ±1000 kb around the gene of interest, coupled with a stringent significance threshold of P < 5.0 × 10^-8^ [20, 21]. In the standard single-SNP SMR analysis, the top genome-wide significant cis-QTL within ±1 Mb of the target gene was selected as the primary instrumental variable to prioritize locally acting molecular QTLs. SNPs with allele frequency differences greater than 0.2 across the LD reference panel, QTL datasets, and outcome GWAS datasets were excluded..." (p. 9).  "...we further utilized a multi-SNP-based SMR approach (--smr-multi), which incorporates all SNPs within a specific QTL probe window (default ±500 kb) that satisfy two criteria: P < 5.0 × 10^-8^ and linkage disequilibrium r^2^ < 0.9 with the top associated SNP [22]." (p. 10).  Additional note: The present manuscript did not explicitly describe a separate weighting scheme for the selected genetic variants. |
|  | c) | Describe the MR estimator (e.g. two-stage least squares, Wald ratio) and related statistics. Detail the included covariates and, in case of two-sample MR, whether the same covariate set was used for adjustment in the two samples | 8–10 | Materials and Methods, Summary-data-based MR Analysis (pp. 8–10).  "Summary-data-based Mendelian randomization (SMR) was performed to evaluate the potential genetically supported associations between genetically regulated molecular traits of ER stress-related genes, including DNA methylation, gene expression, and protein abundance, and GC risk." (p. 8).  "This approach enhanced statistical power when using top cis-QTLs compared with the conventional MR analysis [19]…" (p. 9).  "Besides exploring the potential genetically supported associations between molecular QTLs (mQTLs, eQTLs, and pQTLs) and GC risk, we further explored cross-omics genetic associations, treating mQTLs as exposures and eQTLs as outcomes, as well as eQTLs as exposures and pQTLs as outcomes." (pp. 9–10).  "Building upon the standard SMR framework, we further utilized a multi-SNP-based SMR approach (--smr-multi)…" (p. 10).  "Findings meeting the criteria (P-SMR < 0.05, P-SMR-multi < 0.05, and P-HEIDI > 0.05) were subsequently subjected to colocalization analysis and a composite assessment involving eQTLs, mQTLs, and pQTLs." (p. 10).  Additional note: The analyses were conducted using the SMR and SMR-multi frameworks. Conventional MR estimators such as the Wald ratio or two-stage least squares were not separately applied or reported. Detailed covariate adjustment information for the original QTL and GWAS source datasets, including whether identical covariate sets were used, was not described in the present manuscript. |
|  | d) | Explain how missing data were addressed | 12–13 | Materials and Methods, External Assessment in Gastric Tissue Transcriptome Dataset (p. 12).  "Raw microarray data were processed by mapping probes to gene symbols, removing probes without corresponding gene annotations, and averaging expression values when multiple probes mapped to the same gene." (p. 12).  Materials and Methods, Correlation Analysis of DNA Methylation and Gene Expression in TCGA-STAD (p. 13).  "To ensure strict cross-omics alignment, samples were precisely matched using the first 15 characters of their TCGA barcodes. After excluding samples with missing expression or target probe methylation data, the paired dataset was extracted." (p. 13).  Additional note: No specific missing-data handling procedure for the summary-level SMR analyses was described in the present manuscript. |
|  | e) | If applicable, indicate how multiple testing was addressed | 12 | Materials and Methods, Functional enrichment analysis (p. 12).  "Enrichment P values were adjusted using the Benjamini-Hochberg method, and terms with adjusted P < 0.05 were considered significantly enriched." (p. 12).  Additional note: Multiple-testing-adjusted results were provided in the supplementary tables for the SMR analyses, although the predefined screening criteria were based on P-SMR < 0.05, P-SMR-multi < 0.05, and P-HEIDI > 0.05 rather than on adjusted P values. |
| 7 | **Assessment of assumptions** | Describe any methods or prior knowledge used to assess the assumptions or justify their validity | 9–11 | Materials and Methods, Summary-data-based MR Analysis (pp. 9–10).  "First, for the relevance assumption, genome-wide significant cis-QTLs located within ±1 Mb of the target gene were selected as instrumental variables… Second, for the independence assumption, only cis-acting variants near the target gene were used... Third, for the exclusion restriction assumption, SMR does not rely on the conventional stringent linkage disequilibrium (LD) clumping strategy commonly used in multi-instrument MR. Instead, it leverages the local LD structure within the cis-region and evaluates potential LD-driven associations using the HEIDI test and, where applicable, the SMR-multi extension." (p. 9).  "Subsequently, the HEIDI test was applied to assess heterogeneity potentially attributable to linkage disequilibrium and to help distinguish shared genetic signals from associations driven by distinct linked variants." (p. 10).  Materials and Methods, Colocalization Analysis (pp. 10–11).  "Colocalization analysis was used to identify whether ER stress-related cis-QTLs... and GC GWAS signals were likely to share a common causal variant…" (pp. 10–11). |
| 8 | **Sensitivity analyses and additional analyses** | Describe any sensitivity analyses or additional analyses performed (e.g. comparison of effect estimates from different approaches, independent replication, bias analytic techniques, validation of instruments, simulations) | 7, 9–14 | Materials and Methods, Data Sources (p. 7).  "For replication, we used two independently defined GC phenotypes from the UK Biobank." (p. 7).  Materials and Methods, Summary-data-based MR Analysis (pp. 9–10).  "…we further explored cross-omics genetic associations, treating mQTLs as exposures and eQTLs as outcomes, as well as eQTLs as exposures and pQTLs as outcomes." (pp. 9–10).  "Building upon the standard SMR framework, we further utilized a multi-SNP-based SMR approach (--smr-multi)…" (p. 10).  "Subsequently, the HEIDI test was applied to assess heterogeneity potentially attributable to linkage disequilibrium and to help distinguish shared genetic signals from associations driven by distinct linked variants." (p. 10).  Materials and Methods, Colocalization Analysis (pp. 10–11).  "Colocalization analysis was used to identify whether ER stress-related cis-QTLs (including eQTLs, mQTLs, and pQTLs) and GC GWAS signals were likely to share a common causal variant…" (pp. 10–11).  Materials and Methods, Functional enrichment analysis (pp. 11–12).  "To evaluate whether the gene filtering strategy retained the core biological features of ER stress, functional enrichment analysis was performed separately for the original gene set (2,764 genes) and the final 698-gene set used for SMR analysis…" (pp. 11–12).  Materials and Methods, External Assessment in Gastric Tissue Transcriptome Dataset (p. 12).  "To examine whether the expression patterns of SMR-prioritized candidate genes were directionally consistent with their genetically predicted associations with GC risk, gene expression profiles were obtained from the GEO database…" (p. 12).  Materials and Methods, Correlation Analysis of DNA Methylation and Gene Expression in TCGA-STAD (pp. 12–13).  "To further evaluate the association between specific CpG site methylation and target gene expression, we obtained paired bulk transcriptomic and DNA methylation data from the Stomach Adenocarcinoma (STAD) cohort of The Cancer Genome Atlas (TCGA)…" (pp. 12–13).  Materials and Methods, Single-cell RNA Sequencing Analysis (pp. 13–14).  "To characterize the cell-type-specific distribution of prioritized candidate genes within the gastric tumor microenvironment, single-cell RNA sequencing (scRNA-seq) data (GSE270680) were retrieved from the GEO database…" (pp. 13–14). |
| 9 | **Software and pre-registration** |  |  |  |
|  | a) | Name statistical software and package(s), including version and settings used | 11–14 | Materials and Methods, Colocalization Analysis (p. 11).  "All statistical analyses were performed using R (version 4.3.0). The R packages 'ggplot2' and 'ggrepel' were used to create Manhattan plots, and 'forestplot' was utilized to generate forest plots. Visualization code for SMR locus plots and effect plots (SMRLocusPlot and SMREffectPlot) was adapted from the original SMR implementation described by Zhu et al. [19]." (p. 11).  Materials and Methods, Functional enrichment analysis (pp. 11–12).  "…functional enrichment analysis was performed... using the R package ‘clusterProfiler’." (p. 11).  "Enrichment P values were adjusted using the Benjamini-Hochberg method, and terms with adjusted P < 0.05 were considered significantly enriched." (p. 12).  Materials and Methods, External Assessment in Gastric Tissue Transcriptome Dataset (p. 12).  "Differential expression analysis between GC and normal tissues was performed using the R package 'limma'..." (p. 12).  "For visualization of candidate gene expression, boxplots were generated using the R package ‘ggplot2’..." (p. 12).  Materials and Methods, Correlation Analysis of DNA Methylation and Gene Expression in TCGA-STAD (p. 13).  "All statistical analyses and visualization... were performed in R (version 4.3.3)." (p. 13).  Materials and Methods, Single-cell RNA Sequencing Analysis (pp. 13–14).  "Data processing was performed using the R package 'Seurat' (v5.3.0)." (p. 13).  "...batch correction was performed using the Harmony integration method implemented in the IntegrateLayers function in Seurat. Uniform Manifold Approximation and Projection (UMAP) was then utilized for non-linear dimensionality reduction and visualization using the RunUMAP function based on the top 20 principal components. Cells were clustered using the FindNeighbors and FindClusters functions...." (p. 14). |
|  | b) | State whether the study protocol and details were pre-registered (as well as when and where) | – | Additional note: The study protocol and analysis plan were not pre-registered. |
|  | **RESULTS** |  |  |  |
| 10 | **Descriptive data** |  |  |  |
|  | a) | Report the numbers of individuals at each stage of included studies and reasons for exclusion. Consider use of a flow diagram | 7–8 | Materials and Methods, Study Design (p. 7).  "Figure 1 presents an overview of the study framework and the main analytical procedures." (p. 7).  Materials and Methods, Data Sources (pp. 7–8).  "We obtained the GWAS summary statistics for GC from the FinnGen R10 cohort... including 1,423 cases and 314,193 controls." (p. 7).  "The first was an ICD-10-based phenotype for malignant neoplasm of the stomach (C16), comprising 764 cases and 419,767 controls. The second was based on the UK Biobank self-reported cancer code 1018 (stomach cancer), comprising 155 cases and 420,318 controls." (p. 7).  "Detailed information on all GWAS datasets used in this study is summarized in Supplementary Table S1." (p. 7).  "The original GeneCards-derived genes, the high-priority GeneCards candidates, and the final integrated ER stress-related gene set are provided in Supplementary Table S2." (p. 8).  "We acquired blood eQTL data from the eQTLGen Consortium, encompassing gene expression profiles for 31,684 individuals... The mQTL data were obtained through a meta-analysis of two European cohorts... 614 participants... and 1,366 participants... pQTL data... comprised 54,219 participants..." (p. 8).  "Detailed information on all QTL datasets used in this study is summarized in Supplementary Table S1." (p. 8).  Additional note: As this study was based primarily on publicly available summary-level GWAS and QTL data, individual-level participant flow and reasons for exclusion at each stage were not described in the present manuscript. Figure 1 summarizes the overall analytical workflow rather than individual participant recruitment or exclusion. |
|  | b) | Report summary statistics for phenotypic exposure(s), outcome(s), and other relevant variables (e.g. means, SDs, proportions) | 7–8 | Materials and Methods, Data Sources (pp. 7–8).  "We obtained the GWAS summary statistics for GC from the FinnGen R10 cohort... including 1,423 cases and 314,193 controls." (p. 7).  "The first was an ICD-10-based phenotype for malignant neoplasm of the stomach (C16), comprising 764 cases and 419,767 controls. The second was based on the UK Biobank self-reported cancer code 1018 (stomach cancer), comprising 155 cases and 420,318 controls." (p. 7).  "Detailed information on all GWAS datasets used in this study is summarized in Supplementary Table S1." (p. 7).  "The original GeneCards-derived genes, the high-priority GeneCards candidates, and the final integrated ER stress-related gene set are provided in Supplementary Table S2." (p. 8).  "We acquired blood eQTL data from the eQTLGen Consortium, encompassing gene expression profiles for 31,684 individuals... The mQTL data were obtained through a meta-analysis of two European cohorts... 614 participants... and 1,366 participants... pQTL data... comprised 54,219 participants..." (p. 8).  "Detailed information on all QTL datasets used in this study is summarized in Supplementary Table S1." (p. 8).  Additional note: As this study used publicly available summary-level GWAS and QTL data, detailed individual-level phenotypic summary statistics, such as means, standard deviations, and proportions, were not described in the present manuscript. |
|  | c) | If the data sources include meta-analyses of previous studies, provide the assessments of heterogeneity across these studies | – | Additional note: The mQTL source data were derived from a previously published meta-analysis of two European cohorts. Heterogeneity across the contributing cohorts was not re-assessed in the present study and was not reported in the present manuscript. |
|  | d) | For two-sample MR:  i.  Provide justification of the similarity of the genetic variant-exposure associations between the exposure and outcome samples  ii.  Provide information on the number of individuals who overlap between the exposure and outcome studies | 7–8 | Materials and Methods, Data Sources (pp. 7–8).  "We obtained the GWAS summary statistics for GC from the FinnGen R10 cohort... For replication, we used two independently defined GC phenotypes from the UK Biobank…Detailed information on all GWAS datasets used in this study is summarized in Supplementary Table S1." (p. 7).  "We acquired blood eQTL data from the eQTLGen Consortium... The mQTL data were obtained through a meta-analysis of two European cohorts... Additionally, pQTL data ... comprised 54,219 participants from the UK Biobank [18]. Detailed information on all QTL datasets used in this study is summarized in Supplementary Table S1." (p. 8).  Supplementary Table S1 indicates that the mQTL dataset included European individuals, the eQTL dataset was predominantly of European ancestry, and the pQTL dataset included European-descent individuals. The GC outcome datasets were derived from FinnGen and the UK Biobank.  Additional note: The exposure and outcome datasets were therefore derived predominantly from populations of European ancestry, supporting broad population comparability between the QTL and GC GWAS datasets. However, the exact number of overlapping participants between the exposure and outcome datasets was not reported in the present manuscript or Supplementary Table S1 and could not be determined from the available summary-level information. |
| 11 | **Main results** |  |  |  |
|  | a) | Report the associations between genetic variant and exposure, and between genetic variant and outcome, preferably on an interpretable scale | 15–17 | Results, ER Stress-Related Gene Expression and GC Risk (p. 15).  "In the FinnGen discovery cohort, a total of 10 ER stress-related genes… showed genetic associations with GC risk, as detailed in Supplementary Table S5… the replication analyses using either the UK Biobank C16 or the UK Biobank 1018 GC cohorts, as shown in Supplementary Tables S6 and S7." (p. 15).  Results, ER Stress-Related Gene Methylation and GC Risk (pp. 15–16).  "The complete results of the SMR analysis for ER stress-related mQTL-GWAS can be found in Supplementary Table S8." (p. 15).  "…in the UK Biobank C16 cohort, with the results detailed in Supplementary Table S9… in the UK Biobank 1018 GC cohort, with the corresponding findings presented in Supplementary Table S10." (p. 16).  Results, ER Stress-Related Protein Abundance and GC Risk (p. 16).  "The complete SMR analysis results for ER stress-related pQTL-GWAS are found in Supplementary Table S11... either the UK Biobank C16 or the UK Biobank 1018 GC cohorts, as reported in Supplementary Tables S12 and S13." (p. 16).  Results, Integrating Evidence from Multi-Omics Levels (p. 17).  "These analyses identified genetically supported associations between DNA methylation at key CpG sites and the expression of the corresponding genes… as summarized in Table 1 and Supplementary Table S14." (p. 17).  Supplementary Tables S5–S13 report the top genetic variant for each QTL-GWAS association together with the corresponding genetic variant-exposure and genetic variant-outcome association estimates, including beta coefficients, standard errors, and P values. Supplementary Table S14 similarly reports the top genetic variant together with the corresponding genetic variant-exposure (b_Expo, se_Expo, and p_Expo) and genetic variant-outcome (b_Outco, se_Outco, and p_Outco) association estimates for the cross-omics mQTL-eQTL analyses. |
|  | b) | Report MR estimates of the relationship between exposure and outcome, and the measures of uncertainty from the MR analysis, on an interpretable scale, such as odds ratio or relative risk per SD difference | 15–17 | Results, ER Stress-Related Gene Expression and GC Risk (p. 15).  "The genetically supported associations between the genetically predicted expression levels of key ER stress-related genes and the risk of GC are depicted in Figure 2A. In the FinnGen discovery cohort, a total of 10 ER stress-related genes… showed genetic associations with GC risk, as detailed in Supplementary Table S5... the replication analyses using either the UK Biobank C16 or the UK Biobank 1018 GC cohorts, as shown in Supplementary Tables S6 and S7." (p. 15).  Results, ER Stress-Related Gene Methylation and GC Risk (pp. 15–16).  "The complete results of the SMR analysis for ER stress-related mQTL-GWAS can be found in Supplementary Table S8. A total of 83 CpG sites, corresponding to 53 genes, showed genetically supported associations with GC risk. Figure 2B shows the associations between selected CpG sites and GC risk for genes whose expression levels were also associated with GC risk." (p. 15).  "For instance, the CpG site cg24390590 located near the PCSK6 gene was positively associated with the risk of GC (OR = 1.22, 95% CI = 1–1.49), whereas another CpG site within PCSK6, cg09750144, was negatively associated with GC risk (OR = 0.7, 95% CI = 0.49–0.99)." (pp. 15–16).  "…in the UK Biobank C16 cohort, with the results detailed in Supplementary Table S9… in the UK Biobank 1018 GC cohort, with the corresponding findings presented in Supplementary Table S10." (p. 16).  Results, ER Stress-Related Protein Abundance and GC Risk (p. 16).  "The complete SMR analysis results for ER stress-related pQTL-GWAS are found in Supplementary Table S11. Two ER stress-related proteins, ADIPOQ and YOD1, were identified as negatively associated with GC risk, as illustrated in Figure 2C... either the UK Biobank C16 or the UK Biobank 1018 GC cohorts, as reported in Supplementary Tables S12 and S13." (p. 16).  Results, Integrating Evidence from Multi-Omics Levels (p. 17).  "These analyses identified genetically supported associations between DNA methylation at key CpG sites and the expression of the corresponding genes… as summarized in Table 1 and Supplementary Table S14. The corresponding mQTL-GWAS and eQTL-GWAS signals for these prioritized genes are visualized as Manhattan plots in Figure 3. In contrast, no significant associations were observed between gene expression and protein abundance." (p. 17). |
|  | c) | If relevant, consider translating estimates of relative risk into absolute risk for a meaningful time period | – | Additional note: Absolute risk estimates over a defined time period were not calculated in the present study, as the analyses were based on summary-level QTL and GWAS data and focused on relative association estimates. |
|  | d) | Consider plots to visualize results (e.g. forest plot, scatterplot of associations between genetic variants and outcome versus between genetic variants and exposure) | 15–18 | Results, ER Stress-Related Gene Expression and GC Risk (p. 15).  "The genetically supported associations between the genetically predicted expression levels of key ER stress-related genes and the risk of GC are depicted in Figure 2A." (p. 15).  "Specifically, the colocalization results for CTNNBIP1 are illustrated in Supplementary Figure 2A, with the regional distribution of the GC GWAS and eQTL signals for CTNNBIP1 shown in Supplementary Figure 3A." (p. 15).  Results, ER Stress-Related Gene Methylation and GC Risk (pp. 15–16).  "Figure 2B shows the associations between selected CpG sites and GC risk for genes whose expression levels were also associated with GC risk." (p. 15).  "The colocalization result for cg02833588 corresponding to CTNNBIP1 gene is shown in Supplementary Figure 2B, with the regional distribution of the GC GWAS and mQTL signals for cg02833588 mapped to CTNNBIP1 shown in Supplementary Figure 3B." (p. 16).  Results, ER Stress-Related Protein Abundance and GC Risk (p. 16).  "Two ER stress-related proteins, ADIPOQ and YOD1, were identified as negatively associated with GC risk, as illustrated in Figure 2C." (p. 16).  "The colocalization analysis provided strong evidence for YOD1 (PPH4 > 0.8), which is shown in Supplementary Figure 2C." (p. 16).  Results, Integrating Evidence from Multi-Omics Levels (pp. 17–18).  "The corresponding mQTL-GWAS and eQTL-GWAS signals for these prioritized genes are visualized as Manhattan plots in Figure 3." (p. 17).  "The regional distributions of the GC GWAS and mQTL signals for the selected PCSK6 CpG sites, and of the GC GWAS and eQTL signals for PCSK6, are shown in Supplementary Figure 4A and 4B." (p. 18). |
| 12 | **Assessment of assumptions** |  |  |  |
|  | a) | Report the assessment of the validity of the assumptions | 15–17 | Results, ER Stress-Related Gene Expression and GC Risk (p. 15).  "In the FinnGen discovery cohort, a total of 10 ER stress-related genes… showed genetic associations with GC risk, as detailed in Supplementary Table S5... the replication analyses using either the UK Biobank C16 or the UK Biobank 1018 GC cohorts, as shown in Supplementary Tables S6 and S7. Among the initially identified signals, colocalization analysis provided strong evidence (PPH4 > 0.8) for 1 gene (ATXN3) and suggestive evidence (0.5 < PPH4 ≤ 0.8) for 4 genes (CTNNBIP1, PSMD4, RETREG3, and UFD1)." (p. 15).  Results, ER Stress-Related Gene Methylation and GC Risk (pp. 15–16).  "The complete results of the SMR analysis for ER stress-related mQTL-GWAS can be found in Supplementary Table S8. A total of 83 CpG sites, corresponding to 53 genes, showed genetically supported associations with GC risk." (p. 15).  "Among the initially identified signals, colocalization evidence was observed for 47 sites corresponding to 33 genes. Of these, 9 sites corresponding to 8 genes met the threshold for strong colocalization evidence (PPH4 > 0.8), whereas the remaining sites showed suggestive evidence (0.5 < PPH4 ≤ 0.8)." (pp. 16).  "…in the UK Biobank C16 cohort, with the results detailed in Supplementary Table S9… in the UK Biobank 1018 GC cohort, with the corresponding findings presented in Supplementary Table S10." (p. 16).  Results, ER Stress-Related Protein Abundance and GC Risk (p. 16).  "The complete SMR analysis results for ER stress-related pQTL-GWAS are found in Supplementary Table S11. Two ER stress-related proteins, ADIPOQ and YOD1, were identified as negatively associated with GC risk... ADIPOQ was also supported by suggestive colocalization evidence (0.5 < PPH4 ≤ 0.8)... The colocalization analysis provided strong evidence for YOD1 (PPH4 > 0.8)… either the UK Biobank C16 or the UK Biobank 1018 GC cohorts, as reported in Supplementary Tables S12 and S13." (p. 16).  Results, Integrating Evidence from Multi-Omics Levels (p. 17).  "These analyses identified genetically supported associations between DNA methylation at key CpG sites and the expression of the corresponding genes… as summarized in Table 1 and Supplementary Table S14. In contrast, no significant associations were observed between gene expression and protein abundance." (p. 17).  Supplementary Tables S5–S14 report the corresponding SMR, SMR-multi, and HEIDI test results for the discovery and replication analyses, including P-SMR, P-SMR-multi, and P-HEIDI values. The prioritized discovery signals were selected using the predefined criteria of P-SMR < 0.05, P-SMR-multi < 0.05, and P-HEIDI > 0.05. |
|  | b) | Report any additional statistics (e.g., assessments of heterogeneity across genetic variants, such as *I^2^*, Q statistic or E-value) | 15–17 | Results, ER Stress-Related Gene Expression and GC Risk (p. 15).  "In the FinnGen discovery cohort, a total of 10 ER stress-related genes… showed genetic associations with GC risk, as detailed in Supplementary Table S5... the replication analyses using either the UK Biobank C16 or the UK Biobank 1018 GC cohorts, as shown in Supplementary Tables S6 and S7." (p. 15).  Results, ER Stress-Related Gene Methylation and GC Risk (pp. 15–16).  "The complete results of the SMR analysis for ER stress-related mQTL-GWAS can be found in Supplementary Table S8." (p. 15).  "…in the UK Biobank C16 cohort, with the results detailed in Supplementary Table S9… in the UK Biobank 1018 GC cohort, with the corresponding findings presented in Supplementary Table S10." (p. 16).  Results, ER Stress-Related Protein Abundance and GC Risk (p. 16).  "The complete SMR analysis results for ER stress-related pQTL-GWAS are found in Supplementary Table S11... either the UK Biobank C16 or the UK Biobank 1018 GC cohorts, as reported in Supplementary Tables S12 and S13." (p. 16).  Results, Integrating Evidence from Multi-Omics Levels (p. 17).  "These analyses identified genetically supported associations between DNA methylation at key CpG sites and the expression of the corresponding genes… as summarized in Table 1 and Supplementary Table S14." (p. 17).  Supplementary Tables S5–S14 report the corresponding HEIDI heterogeneity statistics, including P-HEIDI and the number of SNPs included in the HEIDI test (nsnp_HEIDI), together with the SMR and SMR-multi results.  Additional note: Heterogeneity was assessed using the HEIDI test within the SMR framework. Conventional heterogeneity statistics such as I^2^ and Cochran's Q, and E-values, were not calculated in the present study. |
| 13 | **Sensitivity analyses and additional analyses** |  |  |  |
|  | a) | Report any sensitivity analyses to assess the robustness of the main results to violations of the assumptions | 15–17 | Results, ER Stress-Related Gene Expression and GC Risk (p. 15).  "In the FinnGen discovery cohort, a total of 10 ER stress-related genes… showed genetic associations with GC risk, as detailed in Supplementary Table S5... the replication analyses using either the UK Biobank C16 or the UK Biobank 1018 GC cohorts, as shown in Supplementary Tables S6 and S7… colocalization analysis provided strong evidence (PPH4 > 0.8) for 1 gene… and suggestive evidence (0.5 < PPH4 ≤ 0.8) for 4 genes..." (p. 15).  Results, ER Stress-Related Gene Methylation and GC Risk (pp. 15–16).  "The complete results of the SMR analysis for ER stress-related mQTL-GWAS can be found in Supplementary Table S8." (p. 15).  "Among the initially identified signals, colocalization evidence was observed for 47 sites corresponding to 33 genes. Of these, 9 sites corresponding to 8 genes met the threshold for strong colocalization evidence (PPH4 > 0.8), whereas the remaining sites showed suggestive evidence (0.5 < PPH4 ≤ 0.8)." (p. 16).  "…in the UK Biobank C16 cohort, with the results detailed in Supplementary Table S9… in the UK Biobank 1018 GC cohort, with the corresponding findings presented in Supplementary Table S10." (p. 16).  Results, ER Stress-Related Protein Abundance and GC Risk (p. 16).  "The complete SMR analysis results for ER stress-related pQTL-GWAS are found in Supplementary Table S11... ADIPOQ was also supported by suggestive colocalization evidence (0.5 < PPH4 ≤ 0.8)… The colocalization analysis provided strong evidence for YOD1 (PPH4 > 0.8)… either the UK Biobank C16 or the UK Biobank 1018 GC cohorts, as reported in Supplementary Tables S12 and S13." (p. 16).  Results, Integrating Evidence from Multi-Omics Levels (p. 17).  "These analyses identified genetically supported associations between DNA methylation at key CpG sites and the expression of the corresponding genes… as summarized in Table 1 and Supplementary Table S14." (p. 17).  Supplementary Tables S5–S14 report the corresponding single-SNP SMR, SMR-multi, and HEIDI results, including P-SMR, P-SMR-multi, P-HEIDI, and nsnp_HEIDI. Robustness of the main findings was further assessed by colocalization analysis to evaluate whether the QTL and GC GWAS signals were likely to share the same genetic variant, and by independent replication in two UK Biobank GC phenotypes. |
|  | b) | Report results from other sensitivity analyses or additional analyses | 18–21 | Results, External Assessment in a Gastric Cancer Transcriptome Dataset (pp. 18–19).  "Among the six ER stress-related genes overlapping between the SMR results and the differentially expressed genes identified in GSE118916…, three genes… showed consistent expression changes in GSE118916 that aligned with the direction of their SMR associations." (p. 18).  "In contrast, CTNNBIP1…PSMD4… and PPP1R12A… exhibited expression patterns inconsistent with the direction of their genetically predicted associations." (p. 19).  Results, Independent Methylation-Expression Correlation Analysis in the TCGA-STAD Cohort (pp. 19–20).  "The methylation level of cg02833588 exhibited a significant negative correlation with CTNNBIP1 expression in GC tissues (Figure 5A). This observation was directionally consistent with the inverse methylation-expression association suggested by the mQTL-eQTL analysis." (p. 19).  "Regarding PCSK6, we did not observe statistically significant correlations between its mRNA expression and the methylation levels of the candidate CpG probes..." (p. 19).  Results, Cell-type-specific Expression Patterns of Candidate Genes (pp. 20–21).  "…Collectively, these single-cell transcriptomic analyses describe the cell-type-specific expression patterns of PCSK6, YOD1, and RNF5 within the gastric tumor and adjacent normal tissues..." (p. 21). |
|  | c) | Report any assessment of direction of causal relationship (e.g., bidirectional MR) | – | Additional note: Bidirectional MR or other formal directionality analyses were not performed in the present study. The analyses were conducted in prespecified directions, with molecular QTL traits treated as exposures and GC risk as the outcome, and with mQTL to eQTL and eQTL to pQTL directions used in the cross-omics analyses. |
|  | d) | When relevant, report and compare with estimates from non-MR analyses | 18–19 | Results, External Assessment in a Gastric Cancer Transcriptome Dataset (pp. 18–19).  "Among the six ER stress-related genes overlapping between the SMR results and the differentially expressed genes identified in GSE118916…, three genes… showed consistent expression changes in GSE118916 that aligned with the direction of their SMR associations." (p. 18).  "In contrast, CTNNBIP1…PSMD4… and PPP1R12A… exhibited expression patterns inconsistent with the direction of their genetically predicted associations." (p. 19).  Results, Independent Methylation-Expression Correlation Analysis in the TCGA-STAD Cohort (p. 19).  "The methylation level of cg02833588 exhibited a significant negative correlation with CTNNBIP1 expression in GC tissues (Figure 5A). This observation was directionally consistent with the inverse methylation-expression association suggested by the mQTL-eQTL analysis." (p. 19).  Additional note: The non-MR analyses were used as complementary assessments of directional consistency rather than as directly comparable estimates of the same exposure-outcome effect. Therefore, no direct quantitative comparison between MR and non-MR effect estimates was performed. |
|  | e) | Consider additional plots to visualize results (e.g., leave-one-out analyses) | 15–20 | Results, ER Stress-Related Gene Expression and GC Risk (p. 15).  "Specifically, the colocalization results for CTNNBIP1 are illustrated in Supplementary Figure 2A, with the regional distribution of the GC GWAS and eQTL signals for CTNNBIP1 shown in Supplementary Figure 3A." (p. 15).  Results, ER Stress-Related Gene Methylation and GC Risk (p. 16).  "The colocalization result for cg02833588 corresponding to CTNNBIP1 gene is shown in Supplementary Figure 2B, with the regional distribution of the GC GWAS and mQTL signals for cg02833588 mapped to CTNNBIP1 shown in Supplementary Figure 3B." (p. 16).  Results, ER Stress-Related Protein Abundance and GC Risk (p. 16).  "The colocalization analysis provided strong evidence for YOD1 (PPH4 > 0.8), which is shown in Supplementary Figure 2C." (p. 16).  Results, Integrating Evidence from Multi-Omics Levels (p. 18).  "The regional distributions of the GC GWAS and mQTL signals for the selected PCSK6 CpG sites, and of the GC GWAS and eQTL signals for PCSK6, are shown in Supplementary Figure 4A and 4B." (p. 18).  Results, External Assessment in a Gastric Cancer Transcriptome Dataset (p. 19).  "Boxplots of the differential expression results are shown in Figure 4." (p. 19).  Results, Independent Methylation-Expression Correlation Analysis in the TCGA-STAD Cohort (p. 19).  "The methylation level of cg02833588 exhibited a significant negative correlation with CTNNBIP1 expression in GC tissues (Figure 5A)." (p. 19).  "Regarding PCSK6, we did not observe statistically significant correlations between its mRNA expression and the methylation levels of the candidate CpG probes... (Figure 5B-5F)." (p. 19).  Results, Cell-type-specific Expression Patterns of Candidate Genes (p. 20).  "Before integration, PCA visualization showed partially dispersed distributions of cells from different samples in the low-dimensional space (Supplementary Figure 5A). Following Harmony integration, cells from different samples exhibited broader overlap and mixing in the integrated embedding, with less apparent sample-specific separation (Supplementary Figure 5B)." (p. 20).  "Based on the expression profiles of canonical marker genes... we annotated 11 major cell types... (Figure 6A-6C). Descriptive comparison between tumor and adjacent normal tissues showed distinct cell-type-specific expression patterns for the candidate genes (Figure 6D)." (p. 20). |
|  | **DISCUSSION** |  |  |  |
| 14 | **Key results** | Summarize key results with reference to study objectives | 21–22 | Discussion (pp. 21–22).  "In our study, we performed an exploratory multi-omics SMR and colocalization analysis to systematically screen for genetic associations between ER stress-related genes and GC risk... By integrating multi-layered external analyses, including transcriptomic profiling (GSE118916), methylation-expression correlation analysis (TCGA-STAD), and single-cell transcriptomic characterization, we prioritized PCSK6, YOD1, and RNF5 as ER stress-related candidate genes associated with GC risk..." (pp. 21–22). |
| 15 | **Limitations** | Discuss limitations of the study, taking into account the validity of the IV assumptions, other sources of potential bias, and imprecision. Discuss both direction and magnitude of any potential bias and any efforts to address them | 29–31 | Discussion (pp. 29–31).  "However, several limitations must be acknowledged. First, it is important to recognize that SMR analysis is inherently a statistical inference tool based on genetic evidence. Consequently, our findings primarily serve the purpose of gene prioritization rather than providing definitive causal proof. Second... residual horizontal pleiotropy remains an inherent limitation of all MR studies. Moreover, although PPH4 > 0.5 was used as the threshold for exploratory screening... this relatively lenient threshold introduces greater uncertainty than more stringent criteria… Third... the smaller number of GC cases in the UK Biobank cohorts may have limited the statistical power to detect small-to-moderate genetic effects, while differences in population characteristics, genetic background, and phenotype definitions may also have contributed to between-population heterogeneity... non-replication should not be interpreted as conclusive evidence against these associations… Fourth... the current lack of analysis using large-scale stomach-specific eQTL data limits our ability to fully capture tissue-contextual effects… Fifth, regarding data sources, although a larger data release (FinnGen R12) has recently become available, our study was conducted utilizing the R10 release. Sixth, the candidate gene set was constructed by integrating GeneCards-derived ER stress-related genes with two MSigDB gene sets... prioritization based on GeneCards relevance scores may introduce database-dependent bias.... Seventh... we did not integrate spatial transcriptomic or cell-cell communication analyses… Finally, direct functional evidence linking these ER stress-related candidate genes to GC remains scarce... the mechanistic interpretations proposed here should be viewed as working hypotheses rather than established biological mechanisms…" (pp. 29–31). |
| 16 | **Interpretation** |  |  |  |
|  | a) | Meaning: Give a cautious overall interpretation of results in the context of their limitations and in comparison with other studies | 21–31 | Discussion (pp. 21–31).  "By integrating multi-layered external analyses, including transcriptomic profiling (GSE118916), methylation-expression correlation analysis (TCGA-STAD), and single-cell transcriptomic characterization, we prioritized PCSK6, YOD1, and RNF5 as ER stress-related candidate genes associated with GC risk..." (pp. 21-22).  "These findings suggest that the function of PCSK6 may vary across cancer types, cellular compartments, and tumor microenvironmental contexts." (p. 23).  "This suggests that the role of YOD1 may vary across tumor types and biological contexts." (p. 24).  "More broadly, direct GC-specific functional evidence for these prioritized genes remains limited, particularly regarding their involvement in ER stress... Accordingly, the mechanistic interpretations presented above should be considered hypothesis-generating and interpreted in the context of known gene functions together with our multi-omics and single-cell findings." (pp. 27–28).  "A strength of this research is its comprehensive and systematic assessment... Our multi-omics investigation provides a prioritized list of ER stress-related genes associated with gastric cancer risk for subsequent functional investigation…" (pp. 28–29).  "Consequently, our findings primarily serve the purpose of gene prioritization rather than providing definitive causal proof." (p. 29).  "Accordingly, these findings should be interpreted as genetically supported prioritization of candidate genes rather than definitive identification of causal genes..." (p. 30).  "...the mechanistic interpretations proposed here should be viewed as working hypotheses rather than established biological mechanisms." (p. 31). |
|  | b) | Mechanism: Discuss underlying biological mechanisms that could drive a potential causal relationship between the investigated exposure and the outcome, and whether the gene-environment equivalence assumption is reasonable. Use causal language carefully, clarifying that IV estimates may provide causal effects only under certain assumptions | 22–28 | Discussion (pp. 22–28).  "PCSK6 is a member of the proprotein convertase family, which is involved in the activation of a variety of precursor proteins through proteolytic processing [27]. Altered PCSK6 function has been linked to disturbances in protein processing and cellular homeostasis, providing a potential biological connection with ER stress-related processes [28]. Given that ER stress is a well-established contributor to cancer progression, altered PCSK6 expression may affect protein homeostasis and ER stress-related signaling in a context-dependent manner..." (p. 22).  "Mechanistically, previous studies have linked PCSK6 to autophagy-related processes [33]... Additionally, PCSK6 has been shown to modulate the activity of matrix metalloproteinases (MMPs), enzymes known for their role in extracellular matrix degradation and cell invasion, which are hallmarks of cancer metastasis [34]." (p. 23).  "YOD1, with its deubiquitinase activity, is involved in the ERAD pathway, which is crucial for the quality control of proteins within the ER [35]... our study indicated that genetically predicted higher YOD1 protein abundance was associated with lower GC risk, raising the possibility that reduced YOD1 levels may be associated with impaired ERAD-related protein quality control." (p. 24).  "Bioinformatics analysis revealed that the Hippo signaling pathway, crucial for cell proliferation, survival, and apoptosis, is modulated by the cooperative action of YOD1 and USP21 [38, 39]." (pp. 24–25).  "RNF5 is involved in the ERAD pathway, playing a critical role in targeting misfolded proteins for proteasomal degradation [40]... although whether this leads to misfolded protein accumulation or altered stress responses remains to be determined. Moreover, RNF5 has been linked to the regulation of the NF-κB signaling pathway, a critical pathway involved in inflammation and cancer progression [41]." (pp. 25–26).  "Accordingly, the mechanistic interpretations presented above should be considered hypothesis-generating and interpreted in the context of known gene functions together with our multi-omics and single-cell findings." (p. 28).  Additional note: The gene-environment equivalence assumption was not specifically assessed because the exposures were genetically predicted molecular traits rather than conventional environmental exposures. |
|  | c) | Clinical relevance: Discuss whether the results have clinical or public policy relevance, and to what extent they inform effect sizes of possible interventions | 29–32 | Discussion (pp. 29–30).  "Rather than serving as an endpoint, our multi-omics investigation provides a prioritized list of ER stress-related genes associated with gastric cancer risk for subsequent functional investigation." (p. 29).  "Accordingly, these findings should be interpreted as genetically supported prioritization of candidate genes rather than definitive identification of causal genes... Future studies in larger, independent GC GWAS cohorts with more consistent phenotype definitions, together with functional experiments, are needed to further evaluate and replicate these associations and explore the underlying biological mechanisms." (p. 30).  Conclusions (p. 32).  "These findings are hypothesis-generating and provide a basis for further investigation into the biological significance of these candidate genes across different cellular contexts. Although the evidence provided is exploratory, additional functional and mechanistic studies are required to better understand their potential roles in GC." (p. 32).  Additional note: The present study did not evaluate clinical or public policy interventions, and the SMR estimates should not be interpreted as estimates of the expected effect size of modifying these molecular traits therapeutically. Therefore, no intervention effect sizes or direct clinical recommendations were derived from the present analyses. |
| 17 | **Generalizability** | Discuss the generalizability of the study results (a) to other populations, (b) across other exposure periods/timings, and (c) across other levels of exposure | 29–30 | Discussion (pp. 29–30).  "Taken together with the cell-type-specific expression patterns observed here, these findings highlight the heterogeneous cellular distribution of the prioritized genes across epithelial, stromal, and immune compartments." (p. 29).  "Several factors may contribute to this discrepancy, and they are not mutually exclusive: the smaller number of GC cases in the UK Biobank cohorts may have limited the statistical power to detect small-to-moderate genetic effects, while differences in population characteristics, genetic background, and phenotype definitions may also have contributed to between-population heterogeneity." (p. 30).  "Future studies in larger, independent GC GWAS cohorts with more consistent phenotype definitions, together with functional experiments, are needed to further evaluate and replicate these associations and explore the underlying biological mechanisms." (p. 30).  "Fourth, while large-scale blood-based QTL data provided improved statistical power, the current lack of analysis using large-scale stomach-specific eQTL data limits our ability to fully capture tissue-contextual effects." (p. 30).  Additional note: Generalizability to other populations and tissue contexts may therefore be limited. Generalizability across different exposure periods or timings and across different levels of the molecular exposures was not specifically assessed, because the study used genetically predicted molecular traits derived from summary-level QTL data rather than directly measured exposures at defined times or doses. |
|  | **OTHER INFORMATION** |  |  |  |
| 18 | **Funding** | Describe sources of funding and the role of funders in the present study and, if applicable, sources of funding for the databases and original study or studies on which the present study is based | 35 | Funding (p. 35).  "This work was supported by the Joint Program of Science and Technology Program of Liaoning Province (Natural Science Foundation-General Program) (2024-MSLH-274) and Wu Jieping Medical Fund (320.6750.2024-25-13)." (p. 35).  Additional note: The specific role of the funders in the present study was not described in the manuscript. Funding sources for the publicly available databases and original studies used in the present analysis were not separately reported in the present manuscript. |
| 19 | **Data and data sharing** | Provide the data used to perform all analyses or report where and how the data can be accessed, and reference these sources in the article. Provide the statistical code needed to reproduce the results in the article, or report whether the code is publicly accessible and if so, where | 35 | Availability of data and materials (p. 35).  "All data analyzed in this study were obtained from publicly available databases, and the relevant results are included in this article and its supplementary materials." (p. 35).  Additional note: Data sources are referenced throughout the Materials and Methods, with detailed information provided in Supplementary Table S1. The availability of the statistical analysis code was not described in the present manuscript. Readers may contact the corresponding author for further information. |
| 20 | **Conflicts of Interest** | All authors should declare all potential conflicts of interest | 35 | Competing interests (p. 35).  "The authors declare that they have no competing interests." (p. 35). |

This checklist is copyrighted by the Equator Network under the Creative Commons Attribution 3.0 Unported (CC BY 3.0) license.

1.	Skrivankova VW, Richmond RC, Woolf BAR, Yarmolinsky J, Davies NM, Swanson SA, et al. Strengthening the Reporting of Observational Studies in Epidemiology using Mendelian Randomization (STROBE-MR) Statement. JAMA. 2021;326(16):1614-1621.

2.	Skrivankova VW, Richmond RC, Woolf BAR, Davies NM, Swanson SA, VanderWeele TJ, et al. Strengthening the Reporting of Observational Studies in Epidemiology using Mendelian Randomisation (STROBE-MR): Explanation and Elaboration. BMJ. 2021;375:n2233.
